# Structured machine learning modeling to support conservation of deep‐sea benthic biodiversity

**DOI:** 10.1111/cobi.70255

**Published:** 2026-03-10

**Authors:** Gustavo Fonseca, Danilo C. Vieira, Juliane C. Carneiro, Renato S. Carreira, Milena Ceccopieri, Thais N. Corbisier, Adriana Galindo Dalto, Alberto G. Figueiredo, Fabiane Gallucci, Paula Gheller, Simone Brito de Jesus, Helena Passeri Lavrado, Letícia Lazzari, Eduardo Hilzendeger Marcon, Daniel Leite Moreira, Rafael Bendayan de Moura, Ellen Pape, Ana Cláudia Aoki Santarosa, João Regis dos Santos Filho, Silvia Helena de Mello e Sousa, Thaisa Marques Vicente, Luciana Erika Yaginuma, Cinthia Yamashita, Wandrey Watanabe

**Affiliations:** ^1^ Instituto do Mar UNIFESP Santos Brazil; ^2^ Departamento de Química PUC‐Rio Rio de Janeiro Brazil; ^3^ Instituto Oceanográfico USP São Paulo Brazil; ^4^ Departamento de Biologia Marinha, IB UFRJ Rio de Janeiro Brazil; ^5^ Geology and Geophysics Department Fluminense Federal University Niteroi Brazil; ^6^ Marine Biology Research Group Ghent University Ghent Belgium; ^7^ PETROBRAS Research Center Centro de Pesquisas Leopoldo Américo Miguez de Mello (CENPES) Rio de Janeiro Brazil; ^8^ Faculdade de Oceanografia UERJ Rio de Janeiro Brazil

**Keywords:** structure modeling, benthos, continental margin, random forest, environmental essential variables, monitoring, bentos, bosque aleatorio, margen continental, modelo estructural, monitoreo, variables ambientales significativas, 结构建模, 海底生物, 大陆边缘, 随机森林, 环境关键变量, 监测

## Abstract

Biodiversity monitoring programs need to deliver accurate, timely, and actionable predictions. To establish a predictive monitoring program for deep‐sea benthos of the Santos Basin, Brazil, we developed a two‐stage structured model that allowed comparison of biodiversity predictions obtained from environmental simulations (2M‐Sim). We also modeled the environmental variables as a function of spatial and temporal variables and compared this model's predictions with predictions obtained from real environmental data (2M). We built unstructured models (1M) as references to evaluate whether the proposed structured approach was reliable. We expected no significant differences between 1M and 2M or between 2M and 2M‐Sim. Data were obtained from 100 stations at depths of 25–2400 m during two surveys (2019 and 2021). In our model framework, we used 12 benthic macro‐ and meiofaunal variables, 44 sediment and water column environmental variables, and four spatial and temporal variables. We applied a random forest algorithm to the structured and unstructured models. All comparisons were performed with 20% of the dataset set aside for validation. The average accuracy was 72%, 69%, and 68% for the 1M, 2M, and 2M‐Sim models, respectively. Accuracies of 2M ranged from 38% to 84% and were generally higher for macrofauna. The observed accuracy loss from 1M to 2M (3%) and from 2M to 2M‐Sim (1%) was not significant for any biodiversity variable. The 2M model identified 30 significant environmental variables; bottom water parameters and sedimentary phytopigment and carbonate concentrations were the best predictors. Our approach supports biodiversity conservation by optimizing data needs and future sampling and by guiding data‐driven management decisions for benthic biodiversity.

## Abstract (es)

Modelos de aprendizaje mecánico estructurado para apoyar a la conservación de la biodiversidad béntica de las profundidades

**Resumen:**

Los programas de seguimiento de la biodiversidad deben ofrecer predicciones precisas, oportunas y aplicables. Para establecer un programa de seguimiento predictivo del bentos profundo de la cuenca de Santos, en Brasil, desarrollamos un modelo estructurado en dos etapas que permitía comparar las predicciones de biodiversidad obtenidas a partir de simulaciones ambientales (2M‐Sim). También modelamos las variables ambientales en función de variables espaciales y temporales y comparamos las predicciones de este modelo con las obtenidas a partir de datos ambientales reales (2M). Creamos modelos no estructurados (1M) como referencia para evaluar si el enfoque estructurado propuesto era fiable. No esperábamos diferencias significativas entre 1M y 2M ni entre 2M y 2M‐Sim. Los datos se obtuvieron de 100 estaciones a una profundidad de entre 25 y 2400m durante dos estudios (2019 y 2021). En nuestro marco de modelo, utilizamos 12 variables de macrofauna y meiofauna bentónica, 44 variables ambientales de sedimentos y columna de agua, y 4 variables espaciales y temporales. Aplicamos un algoritmo de bosque aleatorio a los modelos estructurados y no estructurados. Todas las comparaciones se realizaron con el 20% del conjunto de datos reservado para la validación. La precisión media fue del 72%, 69% y 68% para los modelos 1M, 2M y 2M‐Sim, respectivamente. Las precisiones de 2M oscilaron entre el 38% y el 84% y, en general, fueron mayores para la macrofauna. La pérdida de precisión observada entre 1M y 2M (3%) y entre 2M y 2M‐Sim (1%) no fue significativa para ninguna variable de biodiversidad. El modelo 2M identificó 30 variables ambientales significativas; los parámetros del agua del fondo y las concentraciones de fitopigmentos y carbonatos sedimentarios fueron los mejores predictores. Nuestro enfoque apoya la conservación de la biodiversidad al optimizar las necesidades de datos y el muestreo futuro, y al orientar las decisiones de gestión basadas en datos para la biodiversidad bentónica.

## 利用结构化机器学习建模支持深海底栖生物多样性保护

**【摘要】** 生物多样性监测计划需提供准确、及时且可操作的预测结果。为建立巴西桑托斯盆地深海底栖生物的预测性监测计划, 本研究开发了双阶段结构化模型, 用于比较环境模拟(2M‐Sim)得到的生物多样性预测结果。我们构建了环境变量与时空变量的函数, 并将该模型预测结果与真实环境数据(2M)预测结果进行比较。此外, 我们还构建了非结构化模型(1M)作为参照, 以评估我们建立的结构化方法的可靠性。我们预期1M与2M之间、2M与2M‐Sim之间不存在显著差异。数据来自2019年与2021年对25‐2400米深的100个站点的两次采样调查。模型框架采用12个底栖大型及小型动物群的生物变量、44个沉积物与水柱环境变量以及4个时空变量。我们将随机森林算法用于结构化与非结构化模型, 并在所有比较中预留20%的数据集用于验证。结果显示, 1M、2M及2M‐Sim模型的平均准确率分别为72%、69%和68%。2M模型的准确率范围为38%至84%, 且在大型动物群预测中普遍更高。从1M到2M模型(3%)及从2M到2M‐Sim模型(1%)的准确率下降程度在所有生物多样性变量中均不显著。2M模型识别出30个重要的环境变量, 其中底层水体参数、沉积物植物色素及碳酸盐浓度为最佳预测因子。本方法通过优化数据需求与未来采样方案, 指导数据驱动的底栖生物多样性管理决策, 以支持生物多样性保护。**【翻译:胡怡思;审校:聂永刚】**

## INTRODUCTION

Conservation aims to protect and manage natural environments by preserving their biodiversity, functionality, and resilience against anthropogenic disturbances. This requires characterization of the ecosystem baseline conditions through observation and analysis of multiple environmental parameters such that natural variability is distinguishable from variability caused by climate and other anthropogenic changes (Linder et al., 2017; Sparrow et al., 2020). Given the complexity of ecosystems and their vulnerability to human pressures, environmental monitoring programs must quickly obtain and process data and make accurate predictions to support effective management decisions. Most environmental monitoring programs collect extensive data but still fail to model them effectively (Lovett et al., 2007; Rowland et al., 2021; Schreiber et al., 2022). Traditionally, ecological studies have modeled biodiversity indicators with all the environmental, spatial, and temporal variables simultaneously (hereafter *unstructured approach*). This approach can be used to determine the proportion of variance explained by each of the three variables (Dray et al., 2012). Its disadvantages include a limited capacity to project biodiversity measures without collecting data on the entire set of predictors and the inability to extrapolate the biodiversity measures to less studied areas or the future (Simmonds et al., 2024). An unstructured approach is ineffective for ecosystem monitoring programs that generally involve multiple biodiversity indicators, need to anticipate unwanted ecosystem changes, and require timely management decisions. An alternative is to use a structured modeling framework, in which the modeling steps are explicitly linked based on cause–effect relationships (Schuwirth et al., 2019). The choice of modeling approach is thus fundamental to making robust inferences related to spatial and temporal patterns, to minimizing the amount of data needed to make inferences, and to guiding the best management decisions (Gerbhardt et al., 2024; Walling & Vaneeckhaute, 2020; Zurell et al., 2022).

A critical aspect to be considered when implementing a model‐oriented ecosystem monitoring program is the amount of information needed to make predictions. Collecting and processing biodiversity data requires significant resources, especially for offshore marine biodiversity (Nigard et al., 2016). Efficient monitoring programs for marine ecosystems should be based on essential environmental variables (EEVs) that are easily obtained and processed and capable of providing accurate predictions of the target variables (Ditria et al., 2022). It includes biological and physicochemical variables that can be obtained remotely or in situ, preferably with sensors to speed data processing (Muller‐Karger et al., 2018). Particularly for the benthos along continental margins, EEVs operate across spatial and temporal scales in pelagic and benthic systems. Priority should be given to those related to food availability, oxygen concentration, and mean grain sizes, which vary with depth and influence the community structure and lead to distinct zonation patterns from shelf to abyssal plains (Levin et al., 2001). Additional EVVs to be considered are related to water masses. Water masses have unique physicochemical signatures that regulate ocean productivity and determine the carbon flux to the seafloor, which has a positive effect on benthic biodiversity (Danovaro et al., 2020; Rafaelli et al., 2003; Smith et al., 2008). Therefore, understanding how these drivers collectively structure biodiversity and which can be used as proxies of target indicators is critical for designing monitoring frameworks that balance the complexity of the oceanographic processes with feasibility, particularly in areas with the potential for extractive industries (Reyers et al., 2017).

We sought to develop a structured modeling framework grounded in a hierarchical framework in which spatial and temporal drivers (e.g., bathymetry, seasons) shape environmental conditions, which in turn regulate the benthic biodiversity. With spatial drivers, structured modeling increases analytical complexity, which can decrease performance relative to an unstructured approach, but it makes data acquisition easier and improves the generalization potential of its predictions (Eisenhauer et al., 2015). Explicitly incorporating this hierarchy allows simulation of environmental conditions based on the spatial and temporal drivers. The results of the simulations can then be used to estimate biodiversity patterns. We focused on the practical trade‐off between model accuracy and monitoring efficiency by specifically testing whether a structured model generates predictions as good as an unstructured model. We also examined whether the performance of a structured model based on the simulation of the environmental conditions is comparable to the performance of a model generated from real data.

We used machine learning (ML) algorithms as the analytical foundation for all models. For oceanography, ML models offer advantages over classical statistical methods, including flexibility in handling various data types, ability to address complex problems and confirm predictions on unseen data, and potential to rank predictors in a rigorous hypothesis testing framework (Fonseca & Vieira, 2023). Unlike classical linear models, which rely on strict statistical assumptions (e.g., linearity, normality, homoscedasticity), the core premise of ML is that the training and test data are exchangeable—that is, the training set is representative of the population from which the test set is drawn. Structured ML approaches advance monitoring methods by demonstrating how simulations rooted in EEVs can streamline conservation efforts without compromising insights.

## METHODS

### Study area and sampling design

The Santos Basin is in southeastern Brazil between the Campos Basin and the Pelotas Basin. It is delimited to the north by Cabo Frio High (22°S) and to the south by Florianopolis High (28.5°S) (Moreira et al., 2023). The basin occupies approximately 350,000 km^2^ and borders four Brazilian states along 271 km of the southeastern coast. It reaches 3000 m water depth in the São Paulo Plateau. The continental shelf is narrower (70 km) in the Cabo Frio region (Rio de Janeiro state, RJ) and wider off the city of Santos (230 km), in São Paulo state (SP). Mean declivity is ∼0.08°, and shelf‐break depth varies from 120 to 180 m (Figueiredo Jr. et al., 2023).

**FIGURE 1:**
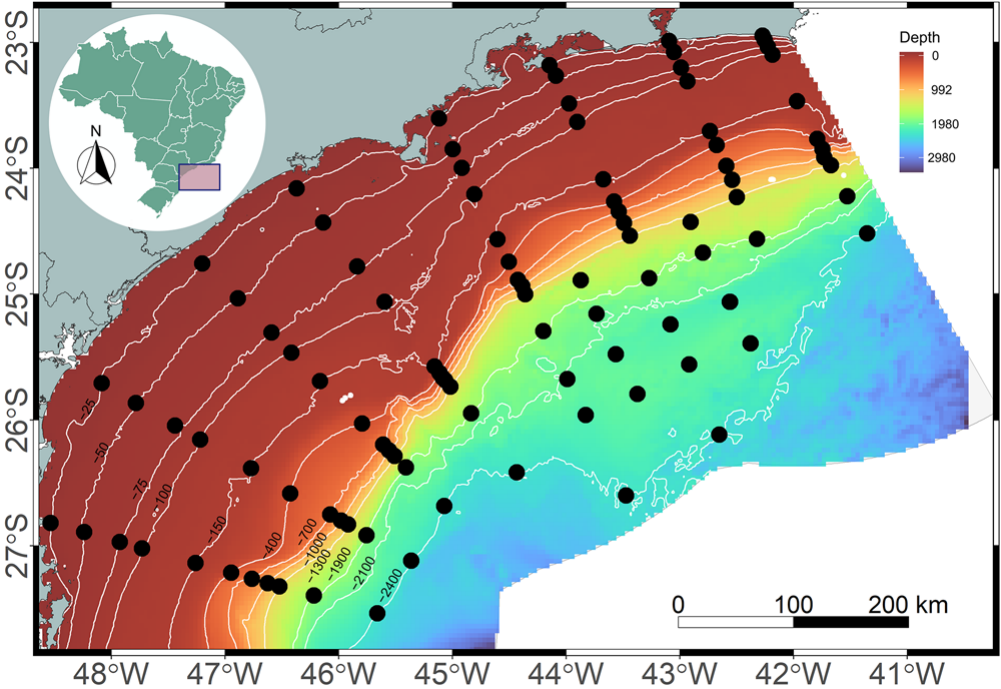
Sampling stations (black dots), isobaths, and the bathymetric color gradient of the Santos Basin, Brazil (grid resolution 2 × 2 km).

Abiotic and biotic benthic data were obtained from the Santos Project (Santos Basin Environmental Characterization) (Moreira et al., 2023). These data were used by the Regional Environmental Characterization Project in support of Petrobras’ Santos Basin drilling licensing process (operation license 1006/2011) led by the Brazilian Environmental Agency (IBAMA). A total of 88 sampling stations were distributed along eight transects perpendicular to the coast and at 11 isobaths (25, 50, 75, 100, 150, 400, 1000, 1300, 1900, and 2400 m) (Figure 1). Twelve additional stations were sampled from 1900 to 2400 m in the São Paulo Plateau, where most of the oil and gas production takes place. Sampling cruises were conducted during 2019 and 2021 (Moreira et al., 2023). Hereafter, we refer to stations at <200 m as being on the continental shelf: 400–700 m, upper slope; 100–1300 m, lower slope; and >1300 m, São Paulo Plateau. In addition to the sampling stations, a 2 × 2‐km bathymetrical grid was generated that had 100,555 data points for each survey.

### Sampling and sample processing

Sediment samples were taken in three replicates with a spade‐type box corer (0.25 m^2^ surface area), or a modified Van Veen grab (231 L, 0.75 m^2^ surface area) was used when the substrate was coarser (sixth station down from the southern‐ and northern‐most transects). To describe the benthos, macrofauna (animals >300 µm) and meiofauna (animals >0.40 µm) were sampled. At each station, macrofauna samples were obtained from nine juxtaposed cylindrical corers of 10 cm^2^ (total 900 cm^2^) inserted in the top 10 cm of the sediment. The meiofauna samples were taken with a cylindrical corer of 5 cm diameter and 10 cm height (19.63 cm^2^ area). All samples were fixed onboard in a 10% formaldehyde solution buffered with borax. Twelve benthic community variables were considered: total macrofauna abundance (individuals/m^2^), meiofauna abundance (individuals/10 cm^2^), macrofauna family richness, meiofauna taxa richness, total macro‐ and meiofaunal polychaeta abundance, total macrofaunal annelid abundance, total abundance of macrofaunal mollusks and crustaceans, and total abundance of meiofaunal nematodes, copepods, and kinorhynchs. A total of 44 environmental variables were analyzed: grain size, parameters of the organic matter, and parameters of the water column 10 m above the bottom (Appendix S1). All the variables listed here are required by IBAMA in the environmental characterization program (IBAMA, 2019). Further details on sampling and analyses of the environmental variables are available in Figueiredo Jr. et al. (2023), Gallucci et al. (2023), Moreira et al. (2023), and Moura et al. (2023).

### Modeling frameworks

We used two distinct modeling frameworks to predict benthic biodiversity patterns from environmental drivers (Appendix S2). This allowed the comparison of a novel structured approach against a traditional unstructured approach commonly used in benthic ecology. The unstructured model (1M) directly linked measured macrofauna and meiofauna data to in situ measurements of environmental variables (organic matter, sediment properties, and bottom water parameters) and geographical coordinates in a single random forest model. In contrast, the structured model had two stages. In the first stage, we developed individual environmental models to predict the spatial distribution of the environmental variables, such as organic matter, sediment, and bottom water parameters. In the second stage, outputs from the first stage—both the original in situ measurements and their model‐predicted (simulated) values—were used as predictors in separate random forest models for biodiversity. This two‐stage design allowed us to generate and compare two distinct types of biodiversity predictions: one based on real environmental data (2M) and the other based on simulated environmental data (2M‐Sim). The latter prediction type allows predictions in areas that lack actual environmental data. We evaluated all models with cross‐validation (CV) and analyzed feature importance (FI) to identify the primary environmental drivers. For FI, all potential predictor variables were input directly to the random forest algorithm. The statistical significance (*p*) of FI was assessed using a permutation‐based approach in which each predictor variable was permuted multiple times to sever its relationship with the response variable. We compared the resulting decay in model performance with the full model (Altman et al., 2010).

### Analytical workflow

Prior to the analyses, replicates were averaged per station to better characterize the local conditions for the purpose of modeling the regional patterns (Gallucci et al., 2023), and biodiversity measures were transformed to log_10_. To enable a comprehensive comparison across the modeling structures and directly quantify how well a model trained on one set of data performs relative to another, unseen set, the dataset was split into two parts: 80% of the data for training models and 20% for model validation (i.e., 38 sampling stations). Model performance was compared using the unseen 20% of the data. Folds were created by randomly splitting the entire dataset (all 100 stations from each survey), not by grouping data by survey or transect. This means that in each iteration of the model training, the training set and the test set contained distinct random subsets of points from both surveys. All models were performed with the random forest regression algorithm. Although we are aware of the importance of testing multiple ML algorithms (Fonseca & Vieira, 2023), we have decided to perform all models with random forest to allow for comparisons across the models. In addition, random forest is generally among the top‐rated models for comparing multiple algorithms (de Jesus et al., 2023; Yaginuma et al., 2025). The parameterization of all the models was the same: a training phase with 500 trees, tune length of 5, a fivefold CV, and five repetitions. The remaining parameters were kept in the default format (Vieira et al., 2025). For each biodiversity model, the FI analysis was performed to retrieve the significant environmental variables selected by the models (*p* < 0.05) and the number at which each variable appeared as a root when all trees were considered. We visualized these results in a Sankey diagram. To verify that the use of random CV did not introduce bias due to spatial autocorrelation and returned over‐fitted models, we tested the spatial independence of residuals from the validation sets with Moran's *I* with *k* nearest‐neighbor spatial weights. Significance was evaluated after applying the Bonferroni correction to account for multiple comparisons across biodiversity descriptors.

### Model comparison

All model comparisons were performed using 20% of the data that were not used in the training phase of the random forest models. First, the differences between the absolute errors of the 1M and 2M were assessed for normality. If the differences were normally distributed, a one‐tailed paired *t*‐test was used under the assumption that significant differences would be detected if the errors increased from 1M to 2M. Otherwise, a paired Wilcoxon test was performed. Such an increase in error can be due to processes related to the effects of time or space on biodiversity not being captured by the environmental variables (Eisenhauer et al., 2015). The second comparison evaluated the differences between the errors of the 2M and 2M‐Sim models, following the same hypothesis testing procedure. Significant changes at this stage would indicate that biodiversity estimates derived from the environmental simulations had higher prediction errors than those based directly on environmental data. All paired tests considered a significant threshold of *p* < 0.05. In addition, a bootstrap resampling procedure (10,000 iterations) was applied to estimate empirical confidence intervals and one‐tailed *p* values. This procedure complemented the paired tests with a nonparametric assessment of model differences.

### Uncertainty propagation analyses

To explicitly quantify the propagation of uncertainty from the environmental simulations to the biodiversity predictions (2M‐Sim), we used a Monte Carlo resampling procedure. For each validation site, we first calculated residuals between observed and simulated environmental predictors obtained from the 2M‐stage models. These residuals represented the discrepancies between measured conditions (used in 2M) and their simulated counterparts (used in 2M‐Sim). In the Monte Carlo procedure, 1000 perturbed versions of the simulated predictors were generated for each site by randomly resampling residuals with replacement. These variable environmental conditions were then propagated through the biodiversity models trained with observed environmental predictors. To account for the intrinsic predictive variability of the biodiversity models, residuals from the biodiversity predictions themselves were also resampled and added to each simulation draw. As a result, each site and descriptor produced an ensemble of predictions that reflected both sources of uncertainty: environmental simulation errors and biodiversity model errors. The resulting ensembles were summarized by their mean, standard deviation, and empirical prediction intervals (PIs) at 90% confidence. To ensure nominal coverage, a conformal calibration step was applied. Absolute errors between observed and mean predictions were used to adjust the interval limits until empirical coverage reached the desired level (Zhou et al., 2025). Interval widths and coverage were then aggregated across descriptors to evaluate and compare the uncertainties across the models.

### Spatial prediction and mapping

Our models generated raster maps in which each pixel's biodiversity value was predicted by a random forest model based on its unique environmental data. These point‐by‐point predictions created spatial patterns based solely on the learned environment–biodiversity relationship. Consequently, areas with similar environmental conditions—regardless of geographic proximity—were predicted to support similar biodiversity.

### Software

We used iMESc (Vieira et al., 2025), an interactive ML application built in R, to perform all data analyses. This platform was selected because it was designed specifically for use with environmental data. It integrates data preprocessing, ML, spatial analysis, and visualization in a reproducible framework. The iMESc application was designed for managing multidisciplinary datasets (e.g., numeric, spatial, categorical) in a unified structure (Datalist), and its dedicated tools for ecological modeling (e.g., Biodiversity tools) and spatial prediction make it uniquely suited for our complex analytical workflow, from model training to the generation of biodiversity prediction maps. The Monte Carlo simulations and conformal calibration procedures for uncertainty propagation were implemented directly in R (R Core Team, 2025). All one‐tailed paired *t*‐tests and Wilcoxon tests were also conducted in R.

## RESULTS

### Model comparisons

The random forest analyses for the 12 descriptors of the fauna in the 1M unstructured model showed that the average model performance was 72% (Table 1). The performance varied from 54% for meiofaunal kinorhynch abundance to 85% for the total abundance of macrofauna. Overall, the macrofauna variables performed better than the meiofauna variables. The accuracy of the structured models (2M) decreased on average by 3% relative to the unstructured 1M (Table 1), but this difference was mostly not significant (Appendices S3–S5). Results from the 2M‐Sim returned, on average, an accuracy of 68%. The best model was for total macrofauna abundance and mollusks (81% accuracy). The paired tests between 2M and 2M‐Sim were all nonsignificant (Appendices S3–S5). Bootstrap resampling confirmed these nonsignificant results, with the exception of Mollusca and Crustacea between 1M and 2M (Appendices S6 & S7). Residuals from the validation sets showed that biodiversity descriptors were not spatially autocorrelated after Bonferroni correction (Moran's *I*) (Appendix S8).

**TABLE 1:** The *R* ^2^ and mean absolute error (MAE) results of regression models of deep‐sea biodiversity variables as a function of the environmental variable for each model structure.

Biodiversity group	Variables	1M^a^		2M^b^		2M‐Sim^c^	
		*R* ^2^	MAE	*R* ^2^	MAE	*R* ^2^	MAE
Meiofauna	n	0.75	0.16	0.71	0.16	0.71	0.17
	nema	0.74	0.16	0.72	0.17	0.70	0.17
	cop	0.66	0.20	0.57	0.22	0.56	0.20
	kino	0.54	0.42	0.46	0.45	0.54	0.43
	poly	0.69	0.23	0.70	0.23	0.67	0.22
	s	0.53	0.04	0.38	0.05	0.44	0.05
Macrofauna	n	0.85	0.12	0.84	0.13	0.81	0.13
	annel	0.81	0.14	0.79	0.14	0.78	0.14
	poly	0.81	0.14	0.80	0.14	0.78	0.14
	crust	0.61	0.22	0.61	0.23	0.56	0.23
	moll	0.82	0.24	0.81	0.24	0.81	0.25
	s	0.83	0.07	0.84	0.07	0.75	0.08
Average		0.72	0.18	0.69	0.18	0.68	0.18

Abbreviations: annel, Annelida; cop, Copepoda; crust, Crustacea; kino, Kinorhyncha; moll, Mollusca; n, abundance; nema, Nematoda; poly, Polychaete; S, taxonomic richness.

^a^ Unstructured model.

^b^ Structured model.

^c^ Structured model with simulations.

The environmental models used to generate predictions of the 2M‐Sim reached, on average, 64% accuracy with the test data (Appendix S1). The models achieved over 80% accuracy for total gravel, very fine gravel, fine sand, and carbonates, and for water temperature, salinity, density, and dissolved oxygen. The best models of organic matter ranged from 78% to 79% accuracy for the chlorophyll‐a‐to‐phaeopigments ratio, total organic matter, nitrogen, and inorganic phosphorus.

### Feature importance

**FIGURE 2:**
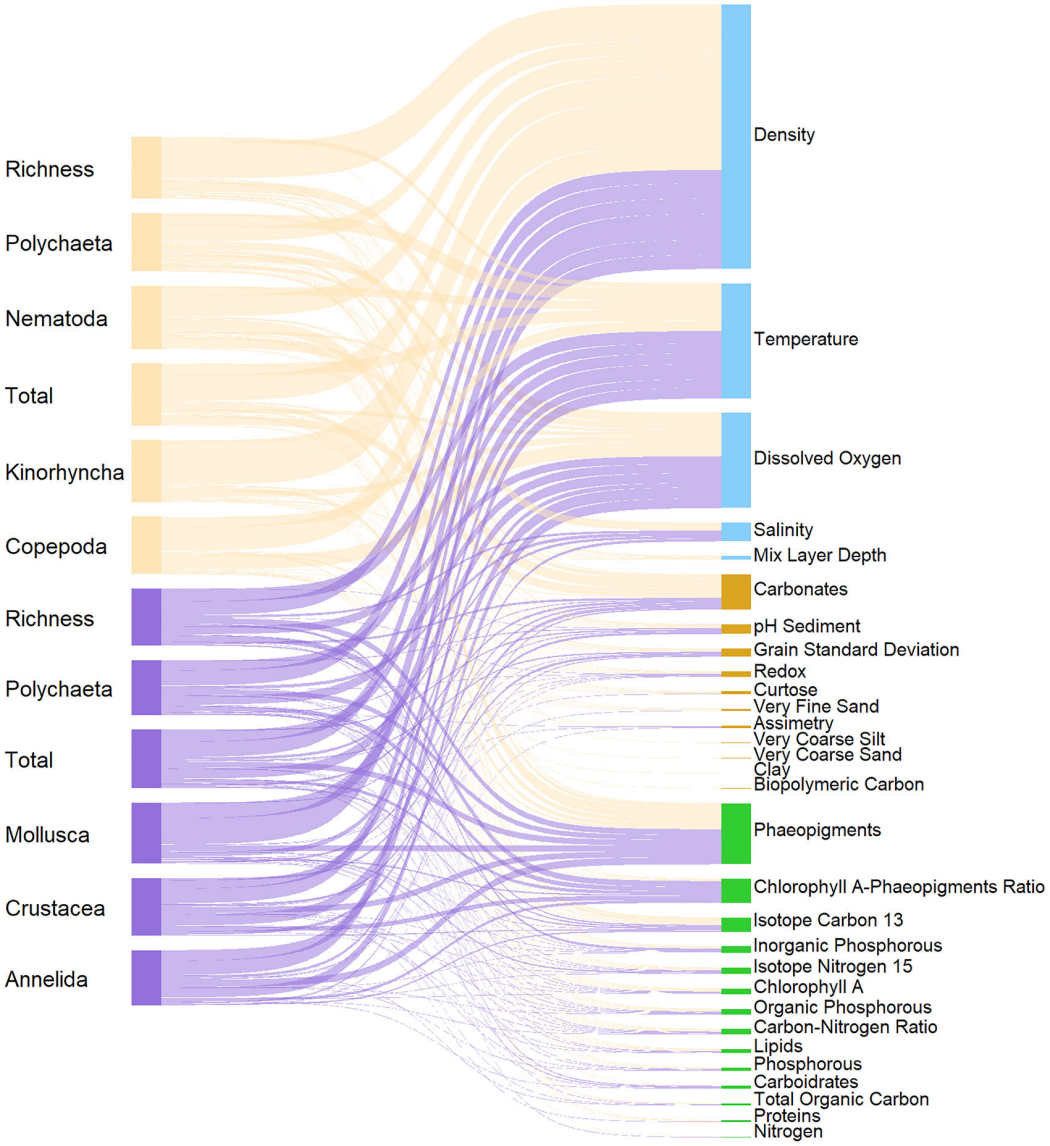
Results of the feature importance analysis for the significant biodiversity variables (*p* < 0.05) of the 2M model (biodiversity predictions obtained from environmental simulations) (link width, proportional to the number of times the variable appeared as the root of the random forest model; node size, relative cumulative importance of each environmental variable across the models; purple nodes, macrofauna; beige nodes, meiofauna; blue nodes, water; green nodes, organic material; yellow nodes, granulometry).

From 44 environmental variables, the FI analysis from the 2M selected 30 variables as significant for at least one benthic community descriptor (Figure 2). Water density, water temperature, and water dissolved oxygen were the three variables that occurred most often in the regression models. The sedimentary chlorophyll‐a‐to‐phaeopigments ratio, concentration of phaeopigments, and percentage of carbonates were also important variables.

### Biodiversity and environmental patterns

The abundance of macrofauna varied on average from 178 to 16,167 individuals per 1 m^2^ (Appendix S9). The number of macrofauna taxa varied on average from 6.3 to 17.3 per station. Polychaetes were the most abundant group of the macrofauna (63%), followed by crustaceans (21%) and mollusks (9%). The abundance of meiofauna varied on average from 29.88 to 2623.89 individuals per 10 cm^2^ (Appendix S9). Nematoda was the dominant group (83%), followed by copepods (12%), polychaetes (2%), and kinorhynchs (1%).

**FIGURE 3:**
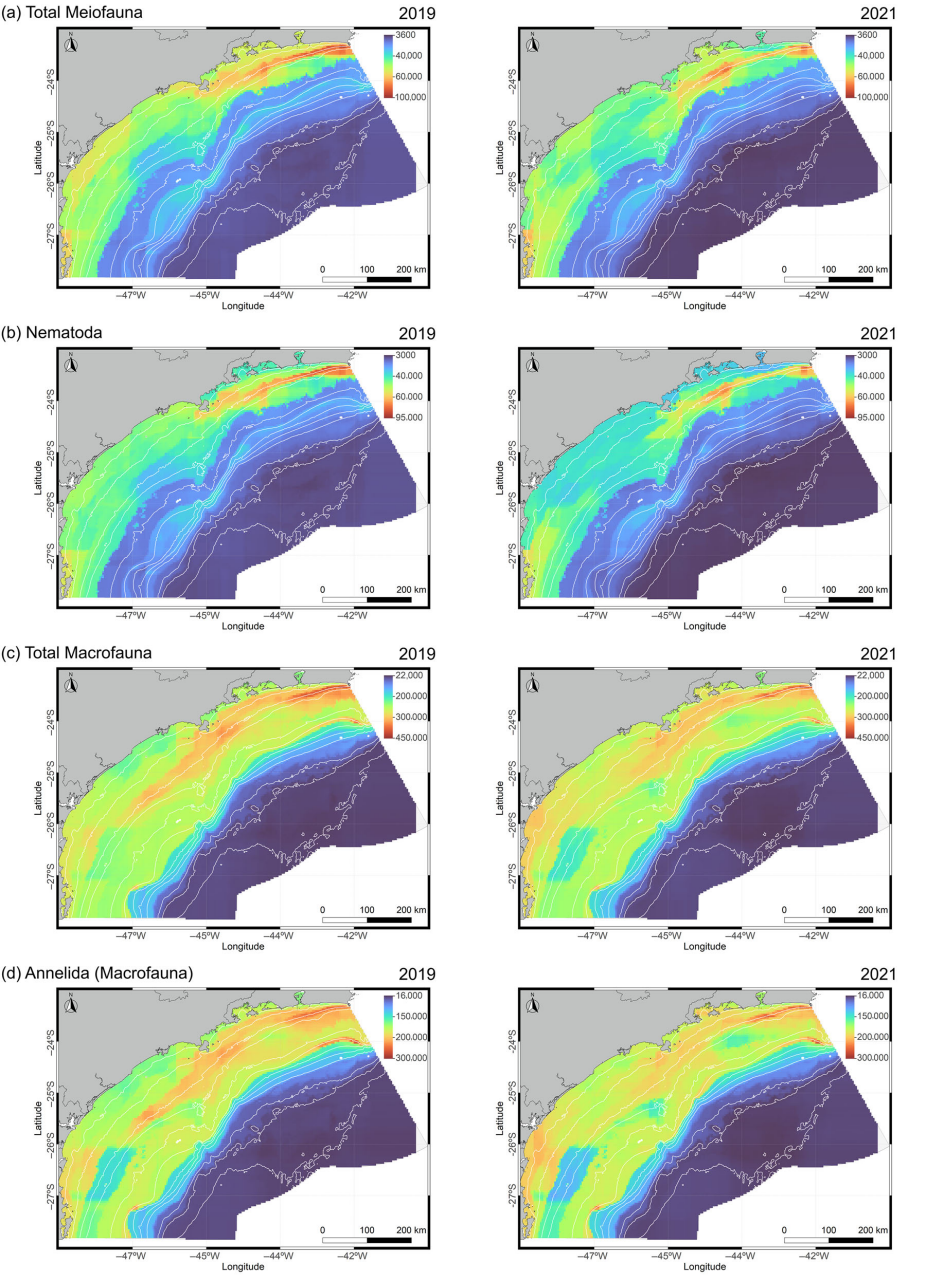
Machine learning model predictions of the abundance of total meiofauna, total macrofauna, Nematoda, and macrofaunal Annelida in the Santos Basin, Brazil, in two survey periods. The remaining maps for biodiversity parameters of meiofauna and macrofauna are in Appendices S10 and S11, respectively.

Meiofauna and nematode abundance were highest down to the shelf break (25–150 m) and at the southern portion of the continental shelf during the first survey (Figure 3). These temporal changes were more evident for meiofauna richness and the abundance of the meiofauna taxa. All parameters of the macrofauna showed a clear bathymetrical gradient; values were highest along the continental shelf and upper slope and lowest at the deeper stations (Figure 3). The richness of the macrofauna was particularly high along the slope compared with the continental shelf. Different from the meiofauna, the macrofauna descriptors did not show clear temporal trends.

**FIGURE 4:**
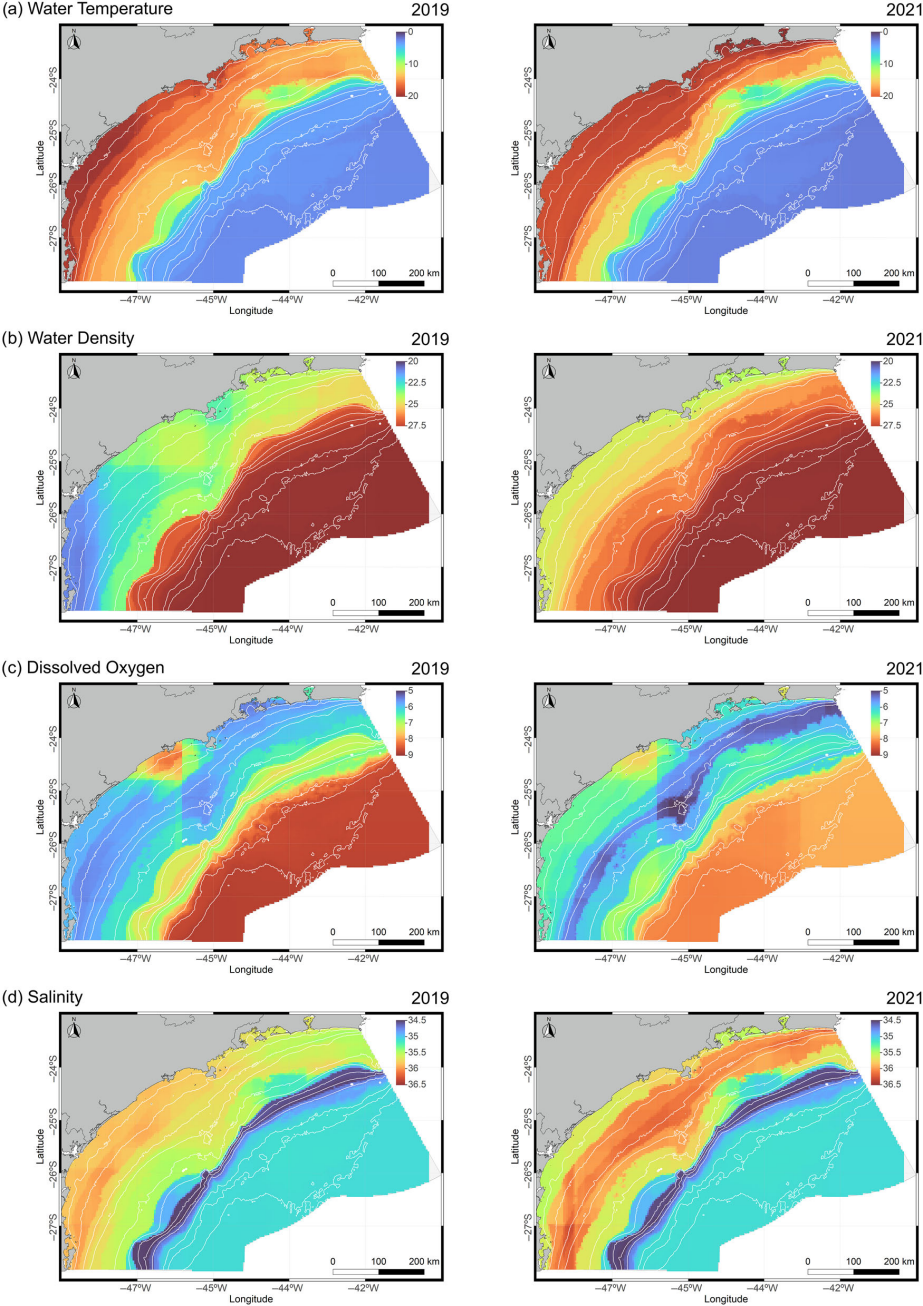
Machine learning model predictions of the most important bottom water parameters (see Figure 3) for predicting the benthic biodiversity variables at the Santos Basin, Brazil, during two surveys. Water density is expressed as density plus 1000 for scaling purposes. Units of the environmental variables are in Appendix S1.

Water density at the deep seafloor was above 1028 kg/m^3^ during both surveys. The southern portion of the continental shelf had much lower water density during the first survey (<1023 kg/m^3^) (Figure 4). Water temperature, dissolved oxygen, and salinity also showed clear spatial and temporal patterns. They all differed among the continental shelf stations, the slope, and São Paulo Plateau stations. Although temperature and salinity varied temporally along the continental shelf, dissolved oxygen varied temporally in the deeper waters.

**FIGURE 5:**
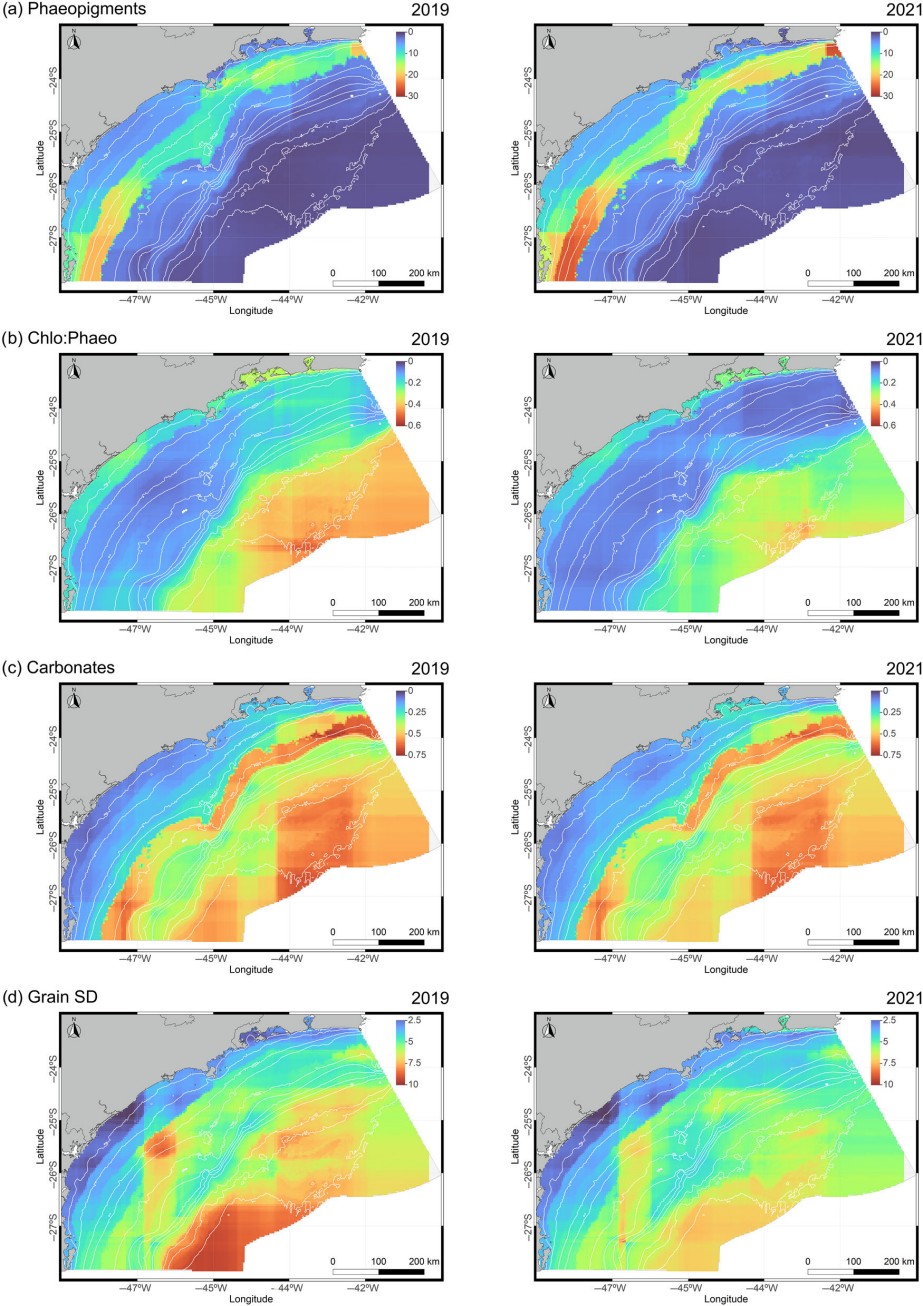
Machine learning model predictions of the most important sediment environmental parameters (see Figure 3) for predicting benthic biodiversity variables at the Santos Basin, Brazil, during two surveys: phaeopigments, (b) Chlo:Phaeo (ratio between chlorophyll‐a and phaeopigments); (c) carbonates, and (d) grain SD (sediment grain sizes standard deviation; units are in Appendix S1).

The concentration of phaeopigments, the chlorophyll‐a‐to‐phaeopigments ratio, and carbonate content in the sediment did not show clear temporal trends but had marked spatial variability (Figure 5). Phaeopigments were higher from 100‐ to 150‐m depths. The chlorophyll‐a‐to‐phaeopigments ratio was highest at the deep‐sea stations, and carbonates were higher at the shelf break and in the deep sea.

### Uncertainty

Results from the Monte Carlo simulations propagated the inherent uncertainty from the simulated environmental predictors into the final biodiversity forecasts. For all biodiversity descriptors, the empirical coverage of the 90% PIs (PI90) met or exceeded the nominal target of 90% (Appendix S12a), confirming that the combined uncertainty from both the environmental simulations and the biodiversity models was accurately quantified. The average width of these PI90 intervals, however, varied among descriptors (Appendix S12b). Wider intervals were associated with kinorhynchs and mollusks, and narrower intervals were observed for richness metrics. At the individual site level, the PIs successfully captured the observed biodiversity values for most descriptors (Appendix S13).

## DISCUSSION

### Oceanographic processes and their influence on spatiotemporal patterns in benthic communities

The oceanographic aspects of the Santos Basin have been investigated thoroughly in recent years in response to industrial interests (Fonseca et al., 2023). Our results confirmed some previous findings. At the basin scale, benthos spatial gradients, especially those related to bathymetry, were much stronger than those related to temporal changes. Temporal fluctuations in biodiversity did occur but were location‐ and process‐specific. For instance, one important oceanographic process at the southeast Brazilian continental shelf was the intrusion of a cold nutrient‐rich water mass with low salinity from the Sub‐Antarctic Argentinian shelf, which brings the runoff from the La Plata River and the Patos Lagoon (Brandini et al., 2018; Sasaki et al., 2024). These waters are carried by the Brazilian Coastal Current, which flows northward and is more intense during austral winters (de Souza & Robinson, 2004). The observed low‐density bottom waters were a strong indication that those waters were present during the sampling in 2019; they reached up to 25°S. The influence of the southern waters promotes primary production in the pelagic system (Brandini, 1990; Brandini et al., 2018), and, as we observed, it sustains a high concentration of phaeopigments in the sediments in both periods. This oceanographic process is indicated mainly by a particular set of benthic biodiversity descriptors. Meiofauna total abundance and taxonomic richness and copepod, kinorhynch, and polychaete abundances were high in the presence of this water mass. It is thus plausible to assume that the observed temporal change in benthic community parameters could be related to their rapid response to organic enrichment, as shown in other studies (Moodley et al., 2005; Witte et al., 2003).

The northern portion of the continental shelf in the Santos Basin is under the influence of coastal upwelling events. During these events, colder and nutrient‐rich South Atlantic Central Water (SACW) rises to the surface, promoting phytoplankton productivity and the deposition of high quantities of organic matter to the bottom (Brandini et al., 2018), which affects benthic systems (De Léo & Pires‐Vanin, 2006; Sumida et al., 2005). The upwelling events are more frequent during austral summers, when northeast winds predominate (Valentin, 2001), and their influence can reach 300 km southward. Despite being perennial on the surface, its effects seem long‐lasting on the seafloor, promoting temporal biodiversity stability (Sumida et al., 2005). During both surveys, higher concentrations of phaeopigments and higher densities of all benthic community parameters were observed at the northern portion of the continental shelf in the Santos Basin relative to the other stations. Additionally, during the surveys, the SACW occupied more than 50% of the water volume in the inner and mid‐shelf of the northern region (Dottori et al., 2023).

At the lower slope (deeper than 400 m) and above the São Paulo Plateau, the parameters of the sediments and water were spatially and temporally homogeneous. Across the Santos Basin, the lower slope was characterized by high concentrations of clay in the sediments and a water mass with low salinity, which is a typical signature of Antarctic Intermediate Water (Stramma & England, 1999). As expected for an oligotrophic and deep‐sea region, very little organic matter and phytodetritus reached the deep‐sea seafloor before or during our surveys. However, during the first survey, the chlorophyll–phaeopigments ratio was higher, suggesting a faster depositional rate of the phytodetritus. This increase in the quality of phytodetritus reaching the seafloor was reflected in higher abundances of all meiofauna taxa and of the macrofaunal crustaceans. As observed for other deep‐sea regions, even small increments of organic material can increase benthic abundances (Billet et al., 1983; Brown et al., 2001).

Meiofauna and nematodes, in particular, occurred in low abundances in sediments with high carbonate content. At the mesophotic shelf break, the main source of bioclastic facies is linked to the presence of bioconstructions, which represent an ecologically important area as a refuge, breeding ground, feeding site, and habitat for benthic and pelagic species (Santos Filho et al., 2022). On the São Paulo Plateau, carbonates are associated with deposits of planktonic shell deposits (mainly pteropods) mixed with hemipelagic muddy terrigenous sediments (Figueiredo Jr. et al., 2023). The larger interstitial space of carbonated sediments does not favor the occurrence of meiofauna groups (Gallucci et al., 2023; Semprucci et al., 2013).

### Applicability of the modeling framework for environmental monitoring programs

We found that applying a structured modeling framework allowed more information to be gathered from the data and made biodiversity measures generalizable across the spatial and temporal scales of the Santos Basin. The lack of significant differences between the structured and unstructured models suggests that the main environmental gradients influencing biodiversity in the region were adequately captured by the modeling framework. Together with the uncertainty analysis, these results confirmed that the structured two‐stage models (2M‐Sim) had a minimum loss of predictive accuracy that was comparable to models based on observed environmental data and provided well‐calibrated estimates of predictive uncertainty. The structured approach allowed identification of cause‐and‐effect relationships among predictors, simulation of the predictions across space and time, and, thus, optimization of data acquisition in a future monitoring program.

By incorporating ML algorithms into the framework, it was possible to select the best model based on the minimum set of EEVs and, most importantly, to evaluate the model's performance on unseen data. Geographical coordinates, bathymetry, time, and water parameters are easily obtained and, at the Santos Basin, can be used to forecast environmental conditions and spatiotemporal benthic community patterns. The next step for implementing a predictive monitoring program that will guide management decisions is to investigate the tipping points of biodiversity in relation to anthropogenic and environmental change (Lewis et al., 2023), keeping in mind that gradual changes and lags in response cannot be discharged (Hillerbrand et al., 2023). These tipping points are important aspects to anticipate and are the basis for optimization of sampling (Henrys et al., 2024).

For the benthos of the Santos Basin, we found that if the 30 environmental variables we investigated are continuously monitored (i.e., simulated and collected to validate the models), biodiversity estimates will be as precise as those obtained from a synoptic sampling. Such findings reinforce the importance of constructing robust analytical frameworks based on sound baseline data for the conservation of marine ecosystems (Gerbhardt et al., 2024; Walling & Vaneeckhaute, 2020; Zurell et al., 2022).

For complex and heterogeneous datasets, it is expected that the biodiversity predictions made from the generalizations of the environmental models (2M‐Sim) will be less precise than those based on the actual data (2M). In theory, this loss can be associated with an error propagation across the modeling structure (Simmonds et al., 2024). Error propagation means that a poor modeling performance in the first stage (e.g., environmental models) will result in low‐quality estimates being used as predictors. However, in our dataset, this propagation was neither constant nor cumulative. The average performance of the environmental models in the 2M modeling structure was relatively low (<56%). Yet, the difference between 2M and 2M‐Sim was only 1% and not significant. This means that even if we improved each environmental model up to 100%, it would still result in a maximum gain of 1% in the biodiversity models. Such findings imply that the current environmental models already capture the major environmental gradients in the basin and that the responses of the biodiversity descriptors to these gradients are probably nonlinear, with critical thresholds (Ehrnesten et al., 2020; Hiddink et al., 2023).

In this context, improvement of the accuracy of the biodiversity models would be achieved by having a larger dataset and by adding other environmental predictors. Additional temporal sampling events to gain more information on spatial and temporal variability will certainly contribute to reducing uncertainty. Incorporating EEVs from the pelagic zone, especially those obtainable through remote sensing, such as surface primary productivity and sedimentation rates (Kavanaugh et al., 2021), might also enhance model performance and speed data acquisition. Another fundamental aspect needed to implement an effective monitoring program is the inclusion of the variables related to the potential disturbances of human activities on the benthos, such as concentrations of metals and hydrocarbons (Nygard et al., 2020). A complete understanding of ecosystem functioning must also consider additional benthic indicators, such as community descriptors of the mega‐ and micro‐benthos (Danovaro et al., 2020; Ingels et al., 2021). These improvements will strengthen understanding of the dynamics at continental margins and the ability to conserve them. Data‐driven monitoring programs, grounded in robust statistical models, are fundamental to keeping pace with the rapid environmental changes induced by human activities.

We used ML to model deep‐sea benthic diversity and thus moved beyond the limitations of classical spatial interpolation (e.g., Kriging), which primarily relies on spatial autocorrelation (Gazis et al., 2024). Our ML structured approach captured the complex, nonlinear relationships between biodiversity and multifaceted environmental drivers (e.g., depth, productivity, sediment properties). This facilitates a shift from purely spatial prediction to process‐based prediction, essential for extrapolating into unsampled areas of similar environmental space (Elith & Leathwick, 2009). Unlike spatial overlay techniques requiring a priori layer weighting (e.g., Xiang, 2001), our model automatically determines the relative importance of each driver and their interactions, providing key ecological insights. The framework is suited for synthesizing multidisciplinary oceanographic datasets (Fonseca & Vieira, 2023). By learning about the environment–response relationship, the ML model makes informed predictions where environmental conditions are within the training range, offering a powerful tool for predicting biodiversity patterns and testing ecological hypotheses.

From a methodological standpoint, the structured modeling framework proved valuable for environmental monitoring programs. The models effectively captured key environmental gradients and enabled the generalization of findings without losing accuracy. Expanding the dataset size and integrating predictors from the pelagic zone could further improve predictive capabilities. The proposed hierarchical approach highlights the interplay between oceanographic processes and benthic communities in the region. Spatial and temporal changes in environmental conditions, such as water masses, sediment properties, and organic matter fluxes, influenced meiofauna and macrofauna distributions. Our results showed that even small increases in phytodetritus in the deep sea led to higher benthic abundance. We concluded that data‐driven monitoring programs, based on structured models and robust baseline data, are crucial for anticipating biodiversity shifts caused by environmental and anthropogenic changes. This approach is critical for the sustainable management and conservation of the Santos Basin ecosystem.

## Supporting information



Supporting Information

## Data Availability

All the data to perform the analyses and the results of the analyses can be assessed as savepoints in the GitHub repository “DaniloCVieira/imesc_savepoints” under the path: https://github.com/DaniloCVieira/imesc_savepoints/tree/1a985ef732eff5e27494ba2b64856a1c9e80870c/Fonseca_2026. The instructions to open the savepoints in iMESc are provided in Appendix S14.
