# Supplementary material for: Structured machine learning modeling to support conservation of deep‐sea benthic biodiversity
Source: Conserv Biol. 2026 Mar 10;40(4):e70255. doi: 10.1111/cobi.70255 (PMC13392758; doi:10.1111/cobi.70255)
Supplement: Supplementary file 1 — Supporting Information [file COBI-40-e70255-s001.docx]

# Supplementary Information

**Appendix S1.** Results of the environmental models used at the structured modeling approach (2M). Sediment parameters: brown background, Organic parameters: green background; water parameters: blue background. PA: presence absence. Abb: Abbreviation

|  |  |  | **Training** | | | | | | **Test** | | |
| --- | --- | --- | --- | --- | --- | --- | --- | --- | --- | --- | --- |
| **Variables** | **Unit** | **Abb** | **R²** | **RMSE** | **MAE** | **RMSESD** | **R²SD** | **MAESD** | **R²** | **RMSE** | **MAE** |
| Redox | - | Redox | 0.56 | 99.95 | 65.75 | 15.49 | 0.13 | 11.19 | 0.51 | 92.85 | 63.15 |
| ph Sediment | - | pH_Sed | 0.61 | 0.18 | 0.14 | 0.03 | 0.11 | 0.02 | 0.56 | 0.16 | 0.12 |
| Median Grain Sizes | µm | Med_Sed | 0.49 | 111.38 | 63.10 | 27.79 | 0.21 | 14.11 | 0.65 | 142.37 | 75.47 |
| Mean Grain Sizes | µm | Mean_Sed | 0.53 | 94.00 | 51.11 | 25.25 | 0.19 | 10.72 | 0.72 | 103.73 | 53.88 |
| Sediment Grain Sizes Standard deviation | µm | Grain SD | 0.43 | 1.48 | 1.17 | 0.15 | 0.09 | 0.12 | 0.48 | 1.27 | 1.03 |
| Assimetry | µm | Assim | 0.49 | 0.16 | 0.12 | 0.02 | 0.09 | 0.01 | 0.59 | 0.17 | 0.13 |
| Curtosis | µm | Curt | 0.27 | 0.31 | 0.21 | 0.06 | 0.14 | 0.04 | 0.62 | 0.23 | 0.15 |
| Total Gravel | % | Tot_Grav | 0.58 | 0.02 | 0.01 | 0.01 | 0.20 | 0.00 | 0.80 | 0.01 | 0.01 |
| Total Sand | % | Tot_Sand | 0.60 | 0.17 | 0.14 | 0.02 | 0.12 | 0.02 | 0.67 | 0.17 | 0.14 |
| Total Mud | % | Tot_Mud | 0.59 | 0.18 | 0.15 | 0.03 | 0.12 | 0.02 | 0.68 | 0.17 | 0.15 |
| Very Fine Gravel | % | VFG | 0.64 | 0.02 | 0.01 | 0.00 | 0.19 | 0.00 | 0.82 | 0.01 | 0.01 |
| Very Coarse Sand | % | VCS | 0.33 | 0.06 | 0.03 | 0.01 | 0.20 | 0.01 | 0.53 | 0.07 | 0.03 |
| Coarse Sand | % | CS | 0.31 | 0.08 | 0.05 | 0.02 | 0.20 | 0.01 | 0.61 | 0.06 | 0.04 |
| Medium Sand | % | MS | 0.26 | 0.08 | 0.05 | 0.02 | 0.14 | 0.01 | 0.56 | 0.06 | 0.05 |
| Fine Sand | % | FS | 0.62 | 0.08 | 0.05 | 0.02 | 0.14 | 0.01 | 0.85 | 0.05 | 0.04 |
| Very Fine Sand | % | VFS | 0.24 | 0.08 | 0.05 | 0.01 | 0.14 | 0.01 | 0.55 | 0.07 | 0.05 |
| Very Coarse Silt | % | VCSilt | 0.61 | 0.03 | 0.02 | 0.01 | 0.11 | 0.00 | 0.52 | 0.04 | 0.03 |
| Coarse Silt | % | Csilt | 0.48 | 0.03 | 0.02 | 0.00 | 0.09 | 0.00 | 0.56 | 0.03 | 0.03 |
| Medium Silt | % | Msilt | 0.49 | 0.04 | 0.03 | 0.01 | 0.14 | 0.00 | 0.66 | 0.04 | 0.03 |
| Fine Silt | % | Fsilt | 0.54 | 0.05 | 0.04 | 0.00 | 0.11 | 0.00 | 0.67 | 0.04 | 0.04 |
| Very Fine Silt | % | VFSIlt | 0.62 | 0.03 | 0.03 | 0.00 | 0.11 | 0.00 | 0.63 | 0.04 | 0.03 |
| Clay | % | Clay | 0.61 | 0.03 | 0.02 | 0.00 | 0.11 | 0.00 | 0.54 | 0.03 | 0.03 |
| Carbonates | % | CBNT | 0.78 | 0.08 | 0.06 | 0.01 | 0.07 | 0.01 | 0.84 | 0.10 | 0.07 |
| Class of Carbonates (lito- or organoclastic) | PA | Class_CBNT | 0.42 | 0.30 | 0.17 | 0.06 | 0.17 | 0.05 | 0.70 | 0.21 | 0.09 |
| Chlorophyl - A | µg/g | Chlo_A | 0.44 | 0.42 | 0.29 | 0.07 | 0.16 | 0.04 | 0.36 | 0.53 | 0.30 |
| Phaeopigments | µg/g | Phaeo | 0.74 | 3.16 | 1.89 | 0.98 | 0.14 | 0.43 | 0.57 | 3.95 | 2.12 |
| Ratio Chlo - A by Phaeo | - | C_P | 0.61 | 0.08 | 0.06 | 0.01 | 0.13 | 0.01 | 0.78 | 0.05 | 0.04 |
| Total Organic Carbon | mg/g | TOC | 0.66 | 0.19 | 0.14 | 0.03 | 0.12 | 0.02 | 0.79 | 0.15 | 0.12 |
| Nitrogen | mg/g | N | 0.57 | 0.03 | 0.02 | 0.01 | 0.14 | 0.00 | 0.78 | 0.03 | 0.02 |
| Ratio Carbon Nitrogen | - | CN | 0.33 | 1.31 | 0.94 | 0.17 | 0.12 | 0.11 | 0.38 | 1.55 | 1.18 |
| Isotope Carbon 13 | ‰ | C13 | 0.51 | 0.51 | 0.36 | 0.11 | 0.15 | 0.07 | 0.63 | 0.48 | 0.33 |
| Isotope Nitrogen 15 | ‰ | N15 | 0.43 | 1.23 | 0.89 | 0.19 | 0.12 | 0.11 | 0.59 | 0.85 | 0.71 |
| Phosphorus Organic | µg/g | P_Org | 0.72 | 60.74 | 48.00 | 6.28 | 0.06 | 4.80 | 0.61 | 77.62 | 62.19 |
| Phosphorus Inorganic | µg/g | P_Inorg | 0.75 | 48.85 | 32.62 | 12.49 | 0.09 | 5.74 | 0.78 | 47.17 | 32.30 |
| Phosphorous Total | µg/g | P_Tot | 0.82 | 76.11 | 56.07 | 16.40 | 0.08 | 10.05 | 0.75 | 101.42 | 79.84 |
| Carbohydrates | mg/g | CHO | 0.62 | 0.40 | 0.30 | 0.06 | 0.10 | 0.03 | 0.63 | 0.38 | 0.29 |
| Proteins | mg/g | PRT | 0.38 | 0.63 | 0.43 | 0.11 | 0.13 | 0.06 | 0.56 | 0.44 | 0.32 |
| Lipids | mg/g | LIP | 0.30 | 0.17 | 0.13 | 0.03 | 0.15 | 0.02 | 0.35 | 0.15 | 0.11 |
| Carbon Biopolymeric | mg/g | CBP | 0.51 | 0.96 | 0.71 | 0.17 | 0.16 | 0.10 | 0.55 | 0.81 | 0.57 |
| Water Temperature | °C | Wtemp | 0.96 | 1.30 | 0.68 | 0.58 | 0.03 | 0.19 | 0.97 | 1.20 | 0.68 |
| Salinity | - | Sal | 0.87 | 0.19 | 0.11 | 0.05 | 0.07 | 0.03 | 0.92 | 0.14 | 0.09 |
| Water Density | kg/m³ | WDens | 0.96 | 0.37 | 0.20 | 0.07 | 0.02 | 0.04 | 0.98 | 0.30 | 0.18 |
| Dissolved Oxygen | mg/L | OD | 0.79 | 0.48 | 0.29 | 0.16 | 0.14 | 0.08 | 0.85 | 0.48 | 0.31 |
| Depth of the Mixed Layer | m | Mix_Lay | 0.69 | 17.68 | 12.79 | 2.35 | 0.10 | 1.56 | 0.10 | 138.91 | 36.12 |
| **Average** |  | **Avg** | **0.56** | **11.90** | **7.72** | **2.49** | **0.13** | **1.36** | **0.64** | **16.33** | **9.38** |


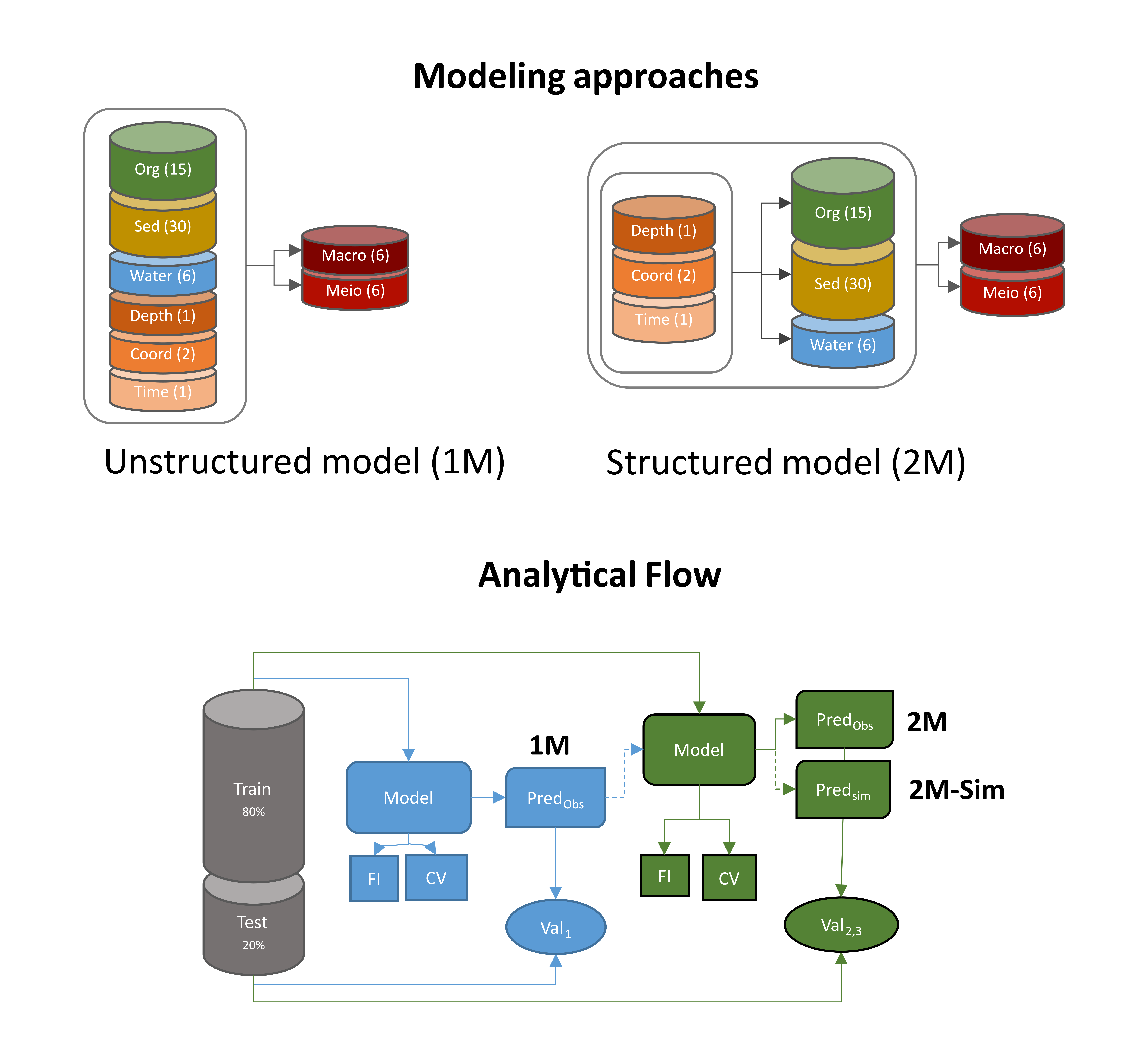
**Appendix S2**

**Appendix S2.** Scheme showing the modeling approaches and analytical flow of the structured model. The analytical flow is separated into two stages: 1) the environmental models are performed (blue icons); 2) the biodiversity models are conducted (green icons). Note that for the biodiversity models, real (full arrow) and predicted (dashed arrow) environmental data are considered generating two types of predictions (2M and 2M-Sim). Org: parameters of organic matter; Sed: parameters of the sediment; Water: parameters of the bottom water; Coord: geographical coordinates; Macro: macrofauna; Meio: meiofauna; FI: feature importance; CV: cross-validation; Pred: Predictions: Obs: Observed values; Sim: simulated values; Val: validation; Env: Environmental. The number between brackets represents the number of variables considered in each group.

**Appendix S3**


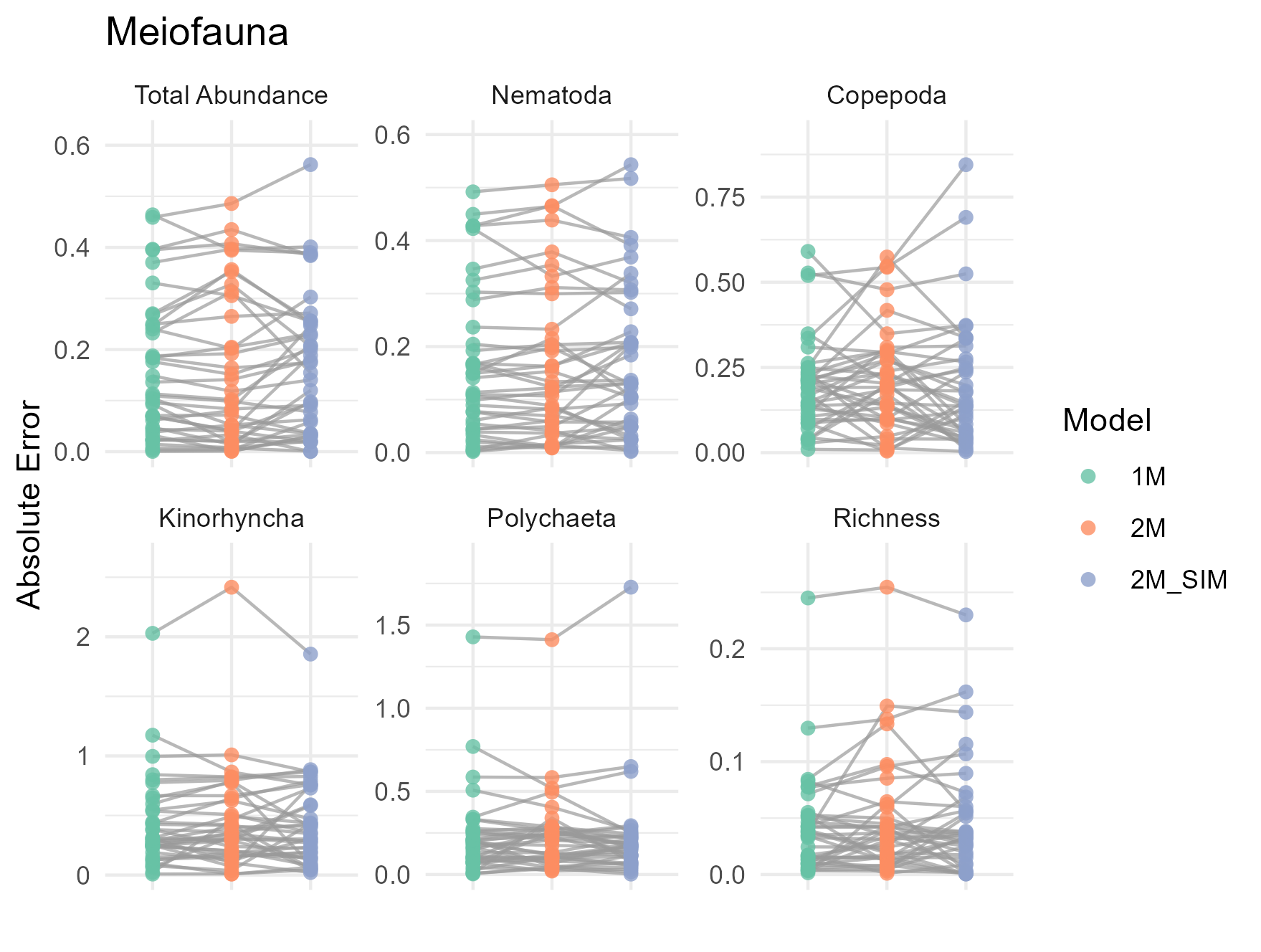


**Appendix S3**. Absolute error of the predictions made for the meiofauna variables from the three modelling approaches (1M, 2M and 2M-Sim).

**Appendix S4**


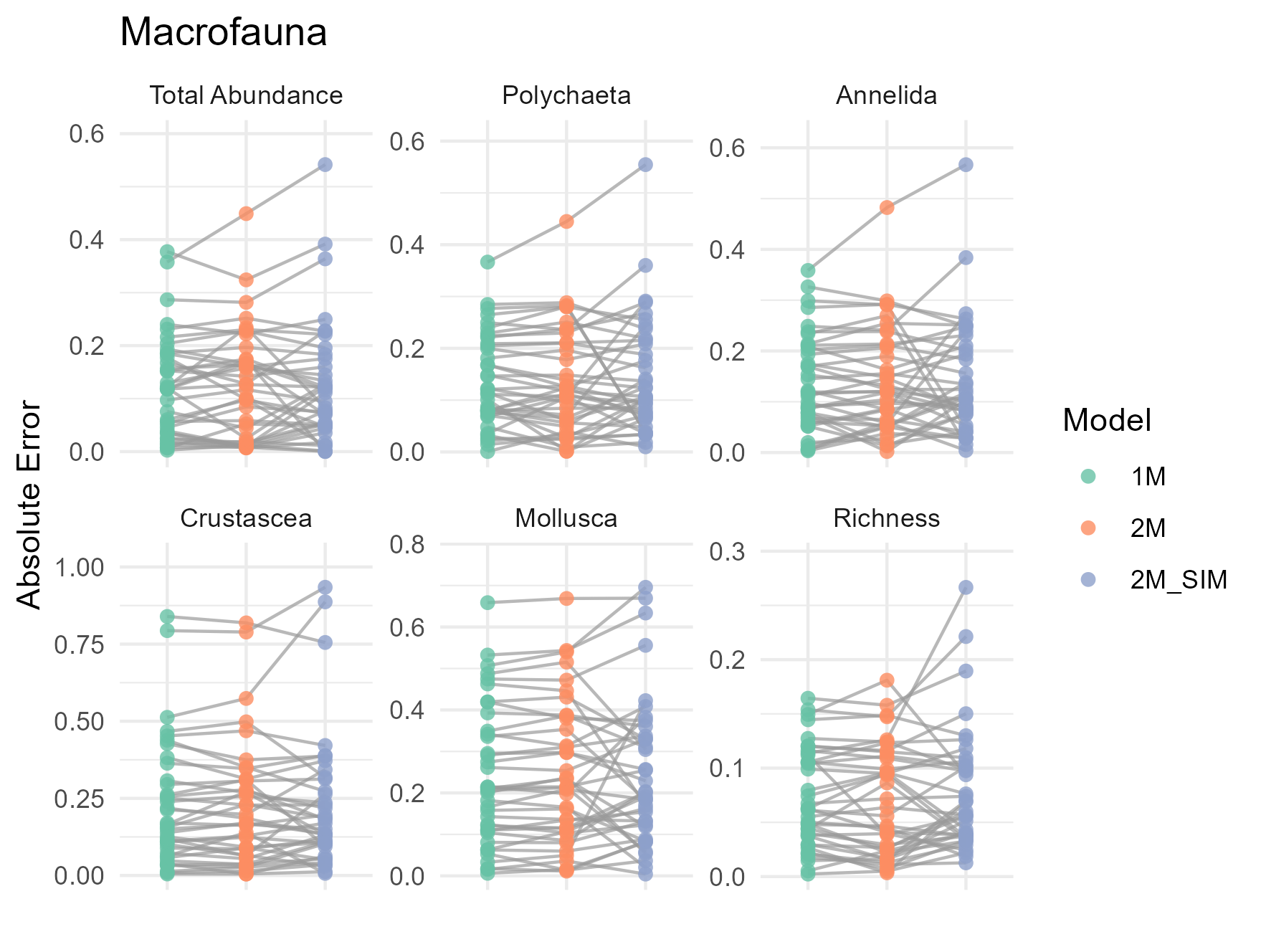


**Appendix S4.** Absolute error of the predictions made for the macrofauna variables from the three modelling approaches (1M, 2M and 2M-Sim).

**Appendix S5.** Results of paired statistical tests comparing prediction errors (Mean Absolute Error, MAE) between models. For each biodiversity descriptor of meiofauna and macrofauna, differences in errors were assessed between the unstructured (1M) and structured (2M) models, and between the structured (2M) and simulation-based (2M-Sim) models. Normality of error differences was first evaluated; when assumptions were met, a one-tailed paired t-test was applied, otherwise a paired Wilcoxon signed-rank test was used. Reported are the test type, test statistic, associated *p*-value (significance threshold set at *p* < 0.05), and effect size (Cohen’s *d* for t-tests, Rosenthal’s *r* for Wilcoxon tests).

| **Comparasion** | **Grupo** | **Model** | **test** | **statistic** | **p.value** | **effect.size** |
| --- | --- | --- | --- | --- | --- | --- |
| 1M vs 2M | Meiofauna | Total Abundance | t.test | -0.09 | 0.46 | -0.01 |
|  |  | Nematoda | wilcox.test | 295.00 | 0.14 | -0.24 |
|  |  | Copepoda | t.test | -0.94 | 0.18 | -0.15 |
|  |  | Kinorhyncha | wilcox.test | 300.00 | 0.16 | -0.23 |
|  |  | Polychaeta | wilcox.test | 379.00 | 0.55 | 0.10 |
|  |  | Richness | wilcox.test | 283.00 | 0.10 | -0.26 |
|  | Macrofauna | Total Abundance | t.test | -0.58 | 0.28 | -0.09 |
|  |  | Annelida | t.test | -0.55 | 0.29 | -0.09 |
|  |  | Polychaeta | t.test | 0.24 | 0.59 | 0.04 |
|  |  | Crustascea | t.test | -0.14 | 0.45 | -0.02 |
|  |  | Mollusca | t.test | -1.46 | 0.08 | -0.24 |
|  |  | Richness | wilcox.test | 431.00 | 0.81 | 0.04 |
| 2M vs 2M_SIM | Meiofauna | Total Abundance | t.test | -1.04 | 0.15 | -0.17 |
|  |  | Nematoda | t.test | -0.85 | 0.20 | -0.14 |
|  |  | Copepoda | t.test | 0.89 | 0.81 | 0.14 |
|  |  | Kinorhyncha | t.test | 0.53 | 0.70 | 0.09 |
|  |  | Polychaeta | t.test | 0.75 | 0.77 | 0.12 |
|  |  | Richness | t.test | 0.29 | 0.61 | 0.05 |
|  | Macrofauna | Total Abundance | t.test | -0.03 | 0.49 | 0.00 |
|  |  | Annelida | wilcox.test | 351.00 | 0.39 | 0.14 |
|  |  | Polychaeta | wilcox.test | 313.00 | 0.21 | -0.21 |
|  |  | Crustascea | t.test | -0.20 | 0.42 | -0.03 |
|  |  | Mollusca | t.test | -0.04 | 0.48 | -0.01 |
|  |  | Richness | t.test | -1.27 | 0.11 | -0.21 |

**Appendix S6**


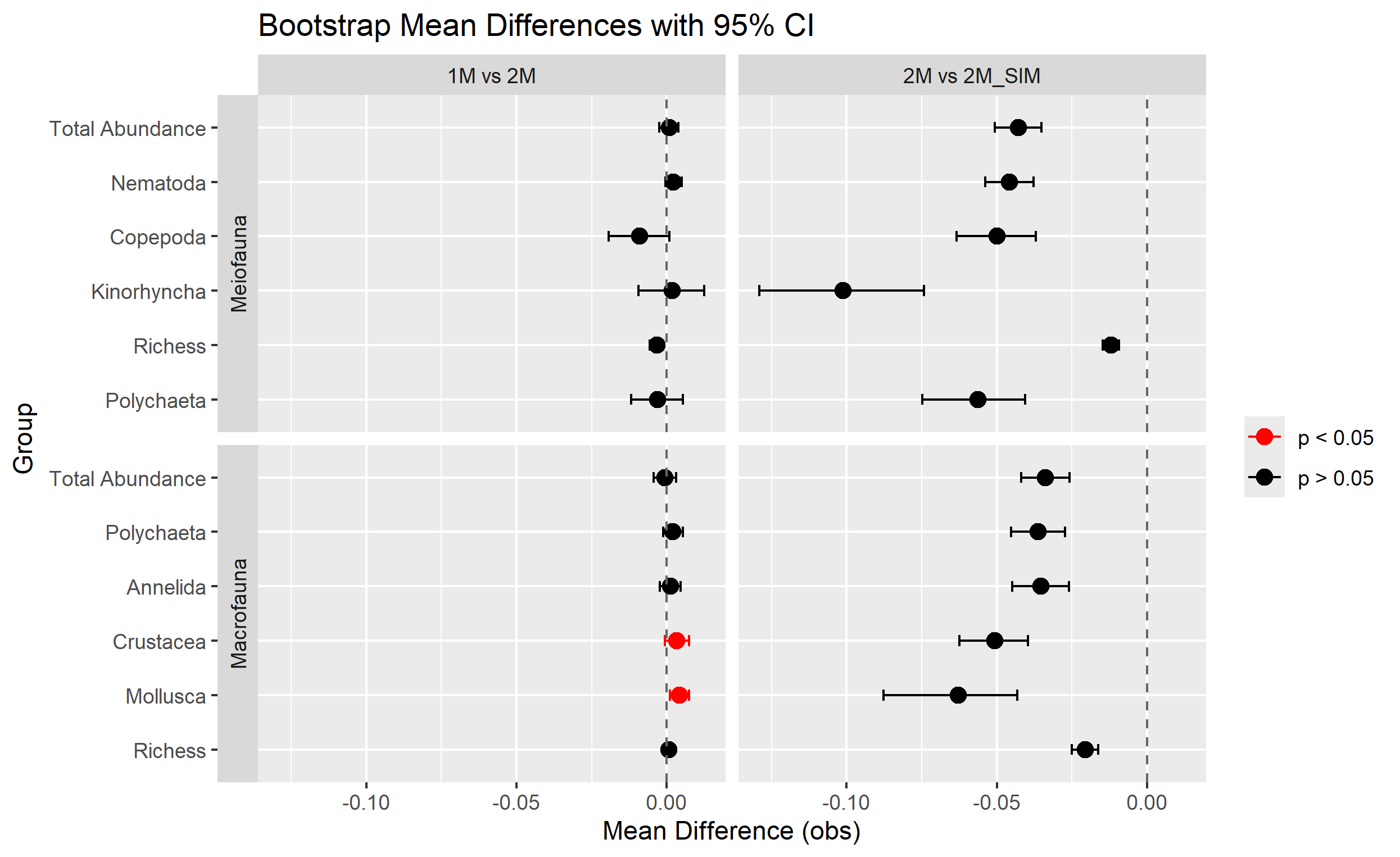


**Appendix S6.** Bootstrap mean differences (95% confidence intervals) between model errors, expressed as Mean Absolute Error (MAE = mean |prediction – observation|). Each point represents the average difference in MAE across sites between two models, with horizontal bars indicating bootstrap confidence intervals (10,000 resamples). Comparisons are shown separately for meiofauna (top panels) and macrofauna (bottom panels), with unstructured vs. structured models (1M – 2M; left panels) and structured vs. simulation-based models (2M – 2M-Sim; right panels). Negative values indicate that the second model in the comparison (2M or 2M-Sim) produced higher errors than the first, whereas positive values indicate the opposite. Red points denote significant results (p < 0.05).

**Appendix S7.** Results of bootstrap resampling analyses (10,000 iterations) comparing model prediction errors expressed as Mean Absolute Error (MAE). For each biodiversity descriptor of meiofauna and macrofauna, the table shows the observed mean difference in MAE (*MAE diff*, which represents the effect size), the 95% bootstrap confidence interval (*c2.5*, *c97.5*), and the one-tailed bootstrap *p*-value (*p* < 0.05 as significance threshold). Comparisons include unstructured vs. structured models (1M vs. 2M) and structured vs. simulation-based models (2M vs. 2M-Sim). Negative values indicate higher MAE for the second model in comparison, while positive values indicate higher MAE for the first.

| **Comparison** | **Group** | **Response** | **MAE diff** | **c2.5** | **c97.5** | **p_value** |
| --- | --- | --- | --- | --- | --- | --- |
| 1M vs 2M | Meiofauna | Total Abundance | 0.00 | 0.00 | 0.00 | 0.31 |
|  |  | Nematoda | 0.00 | 0.00 | 0.00 | 0.05 |
|  |  | Copepoda | -0.01 | -0.02 | 0.00 | 0.96 |
|  |  | Kinorhyncha | 0.00 | -0.01 | 0.01 | 0.37 |
|  |  | Polychaeta | 0.00 | -0.01 | 0.01 | 0.76 |
|  |  | Richness | 0.00 | -0.01 | 0.00 | 1.00 |
|  | Macrofauna | Total Abundance | 0.00 | 0.00 | 0.00 | 0.63 |
|  |  | Annelida | 0.00 | 0.00 | 0.00 | 0.25 |
|  |  | Polychaeta | 0.00 | 0.00 | 0.01 | 0.11 |
|  |  | Crustascea | 0.00 | 0.00 | 0.01 | 0.05 |
|  |  | Mollusca | 0.00 | 0.00 | 0.01 | 0.00 |
|  |  | Richness | 0.00 | 0.00 | 0.00 | 0.17 |
| 2M vs 2M_SIM | Meiofauna | Total Abundance | -0.04 | -0.05 | -0.04 | 1.00 |
|  |  | Nematoda | -0.05 | -0.05 | -0.04 | 1.00 |
|  |  | Copepoda | -0.05 | -0.06 | -0.04 | 1.00 |
|  |  | Kinorhyncha | -0.10 | -0.13 | -0.07 | 1.00 |
|  |  | Polychaeta | -0.06 | -0.07 | -0.04 | 1.00 |
|  |  | Richness | -0.01 | -0.01 | -0.01 | 1.00 |
|  | Macrofauna | Total Abundance | -0.03 | -0.04 | -0.03 | 1.00 |
|  |  | Annelida | -0.04 | -0.04 | -0.03 | 1.00 |
|  |  | Polychaeta | -0.04 | -0.05 | -0.03 | 1.00 |
|  |  | Crustascea | -0.05 | -0.06 | -0.04 | 1.00 |
|  |  | Mollusca | -0.06 | -0.09 | -0.04 | 1.00 |
|  |  | Richness | -0.02 | -0.03 | -0.02 | 1.00 |

**Appendix S8.** Moran’s I test for spatial autocorrelation of model residuals across the Santos Basin. Columns indicate model type, biodiversity descriptor (Y), sampling campaign (2019 and 2021), standard deviate (sd), Moran’s I statistic under the randomization assumption, raw p-values, and Bonferroni-adjusted p-values (p.bonf). The Moran’s I statistic itself represents the magnitude of the spatial autocorrelation effect (i.e., the effect size), with values close to 0 indicating spatial randomness, positive values indicating spatial clustering, and negative values indicating spatial dispersion.

| **Model** | **Y** | **Campaign** | **sd** | **Moran I** | **p.value** | **p.bonf** |
| --- | --- | --- | --- | --- | --- | --- |
| 2M | Total Meiofauna | 2019 | -0.06 | -0.06 | 0.52 | 1.00 |
| 2M | Nematoda (Meiofauna) | 2019 | -0.38 | -0.10 | 0.65 | 1.00 |
| 2M | Copepoda (Meiofauna) | 2019 | 2.39 | 0.19 | 0.01 | 0.40 |
| 2M | Kinorhyncha (Meiofauna) | 2019 | 1.01 | 0.05 | 0.16 | 1.00 |
| 2M | Polychaeta (Meiofauna) | 2019 | -2.10 | -0.27 | 0.98 | 1.00 |
| 2M | Richness (Meiofauna) | 2019 | -0.82 | -0.14 | 0.79 | 1.00 |
| 2M | Total Macrofauna | 2019 | -1.05 | -0.17 | 0.85 | 1.00 |
| 2M | Annelida (Macrofauna) | 2019 | -0.98 | -0.16 | 0.84 | 1.00 |
| 2M | Polychaeta (Macrofauna) | 2019 | -0.81 | -0.15 | 0.79 | 1.00 |
| 2M | Crustascea (Macrofauna) | 2019 | -1.00 | -0.16 | 0.84 | 1.00 |
| 2M | Mollusca (Macrofauna) | 2019 | 0.04 | -0.05 | 0.48 | 1.00 |
| 2M | Richness (Macrofauna) | 2019 | -0.75 | -0.14 | 0.77 | 1.00 |
| 2M | Total Meiofauna | 2021 | -0.17 | -0.07 | 0.57 | 1.00 |
| 2M | Nematoda (Meiofauna) | 2021 | -0.02 | -0.06 | 0.51 | 1.00 |
| 2M | Copepoda (Meiofauna) | 2021 | 0.47 | -0.01 | 0.32 | 1.00 |
| 2M | Kinorhyncha (Meiofauna) | 2021 | 0.46 | -0.02 | 0.32 | 1.00 |
| 2M | Polychaeta (Meiofauna) | 2021 | 1.56 | 0.07 | 0.06 | 1.00 |
| 2M | Richness (Meiofauna) | 2021 | 0.49 | -0.01 | 0.31 | 1.00 |
| 2M | Total Macrofauna | 2021 | -0.35 | -0.09 | 0.64 | 1.00 |
| 2M | Annelida (Macrofauna) | 2021 | 1.76 | 0.12 | 0.04 | 1.00 |
| 2M | Polychaeta (Macrofauna) | 2021 | 1.40 | 0.09 | 0.08 | 1.00 |
| 2M | Crustascea (Macrofauna) | 2021 | 1.35 | 0.08 | 0.09 | 1.00 |
| 2M | Mollusca (Macrofauna) | 2021 | 1.18 | 0.07 | 0.12 | 1.00 |
| 2M | Richness (Macrofauna) | 2021 | -0.89 | -0.15 | 0.81 | 1.00 |
| 2M_SIM | Total Meiofauna | 2019 | -0.81 | -0.14 | 0.79 | 1.00 |
| 2M_SIM | Nematoda (Meiofauna) | 2019 | -1.07 | -0.17 | 0.86 | 1.00 |
| 2M_SIM | Copepoda (Meiofauna) | 2019 | 1.44 | 0.09 | 0.07 | 1.00 |
| 2M_SIM | Kinorhyncha (Meiofauna) | 2019 | -0.43 | -0.10 | 0.67 | 1.00 |
| 2M_SIM | Polychaeta (Meiofauna) | 2019 | -1.47 | -0.22 | 0.93 | 1.00 |
| 2M_SIM | Richness (Meiofauna) | 2019 | -1.39 | -0.20 | 0.92 | 1.00 |
| 2M_SIM | Total Macrofauna | 2019 | -0.48 | -0.11 | 0.68 | 1.00 |
| 2M_SIM | Annelida (Macrofauna) | 2019 | 0.83 | 0.04 | 0.20 | 1.00 |
| 2M_SIM | Polychaeta (Macrofauna) | 2019 | 0.99 | 0.05 | 0.16 | 1.00 |
| 2M_SIM | Crustascea (Macrofauna) | 2019 | -0.87 | -0.14 | 0.81 | 1.00 |
| 2M_SIM | Mollusca (Macrofauna) | 2019 | -0.43 | -0.10 | 0.67 | 1.00 |
| 2M_SIM | Richness (Macrofauna) | 2019 | 0.36 | -0.02 | 0.36 | 1.00 |
| 2M_SIM | Total Meiofauna | 2021 | 0.49 | 0.00 | 0.31 | 1.00 |
| 2M_SIM | Nematoda (Meiofauna) | 2021 | 0.73 | 0.02 | 0.23 | 1.00 |
| 2M_SIM | Copepoda (Meiofauna) | 2021 | 0.47 | -0.01 | 0.32 | 1.00 |
| 2M_SIM | Kinorhyncha (Meiofauna) | 2021 | 0.19 | -0.04 | 0.42 | 1.00 |
| 2M_SIM | Polychaeta (Meiofauna) | 2021 | 1.84 | 0.08 | 0.03 | 1.00 |
| 2M_SIM | Richness (Meiofauna) | 2021 | 1.44 | 0.07 | 0.07 | 1.00 |
| 2M_SIM | Total Macrofauna | 2021 | -1.05 | -0.15 | 0.85 | 1.00 |
| 2M_SIM | Annelida (Macrofauna) | 2021 | 1.00 | 0.03 | 0.16 | 1.00 |
| 2M_SIM | Polychaeta (Macrofauna) | 2021 | 0.74 | 0.01 | 0.23 | 1.00 |
| 2M_SIM | Crustascea (Macrofauna) | 2021 | 0.25 | -0.03 | 0.40 | 1.00 |
| 2M_SIM | Mollusca (Macrofauna) | 2021 | -0.35 | -0.09 | 0.64 | 1.00 |
| 2M_SIM | Richness (Macrofauna) | 2021 | 0.33 | -0.03 | 0.37 | 1.00 |

**Appendix S9.** Minimum, Mean and Maximum average values per station observed for each community descriptor at the Santos Basin, by survey campaign. N represents the total abundance and S the taxonomic richness.

|  | |  | **2019** |  | **2021** | | |
| --- | --- | --- | --- | --- | --- | --- | --- |
| **Variables** | | **Min** | **Mean** | **Max** | **Min** | **Mean** | **Max** |
| Meiofauna | N | 54.66 | 600.71 | 2001.19 | 29.88 | 482.12 | 2623.89 |
|  | Nematoda | 40.23 | 510.90 | 1758.43 | 27.84 | 390.33 | 2260.93 |
|  | Copepoda | 7.64 | 60.97 | 599.95 | 1.36 | 65.41 | 596.04 |
|  | Kinorhynca | 0.00 | 4.10 | 35.31 | 0.00 | 4.36 | 68.59 |
|  | Polychaeta | 0.00 | 10.66 | 70.79 | 0.00 | 10.90 | 186.06 |
|  | S | 8.00 | 11.89 | 16.67 | 6.33 | 10.44 | 17.33 |
| Macrofauna | N | 244.67 | 3271.93 | 13574.00 | 178.00 | 3181.38 | 16166.67 |
|  | Annellida | 185.33 | 2135.49 | 10429.67 | 139.00 | 2103.97 | 12685.00 |
|  | Polychaeta | 181.67 | 2071.46 | 10307.33 | 133.50 | 2043.83 | 12529.67 |
|  | Crustacea | 25.67 | 633.95 | 3458.33 | 16.50 | 609.43 | 6075.00 |
|  | Mollusca | 3.67 | 305.19 | 2222.00 | 0.00 | 246.89 | 1483.33 |
|  | S | 14.00 | 40.25 | 77.33 | 10.50 | 37.95 | 73.67 |

**Appendix S10**


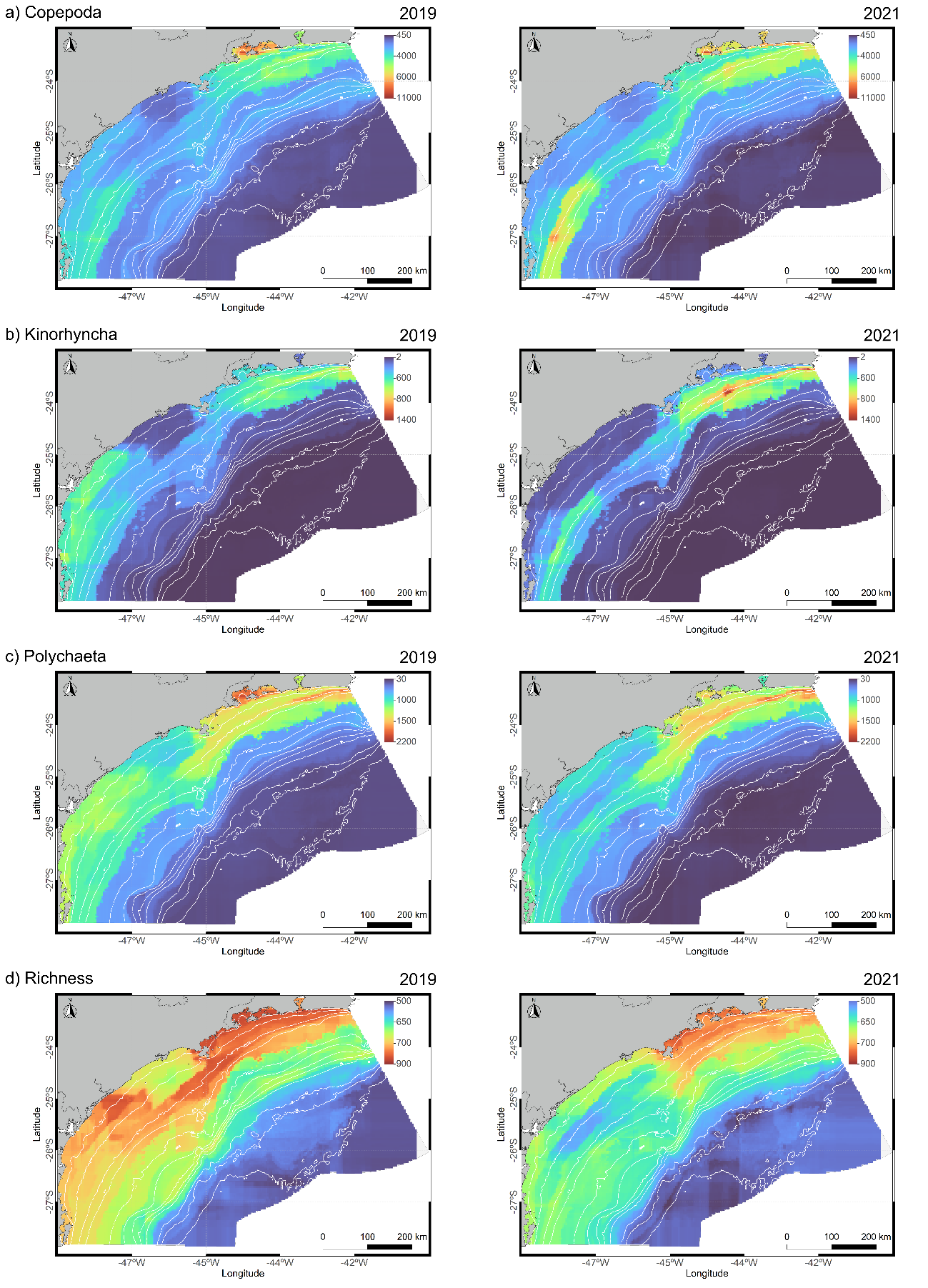


**Figure S10.** Model predictions of the meiofauna at the Santos Basin during both sampling campaigns.

**Appendix S11**


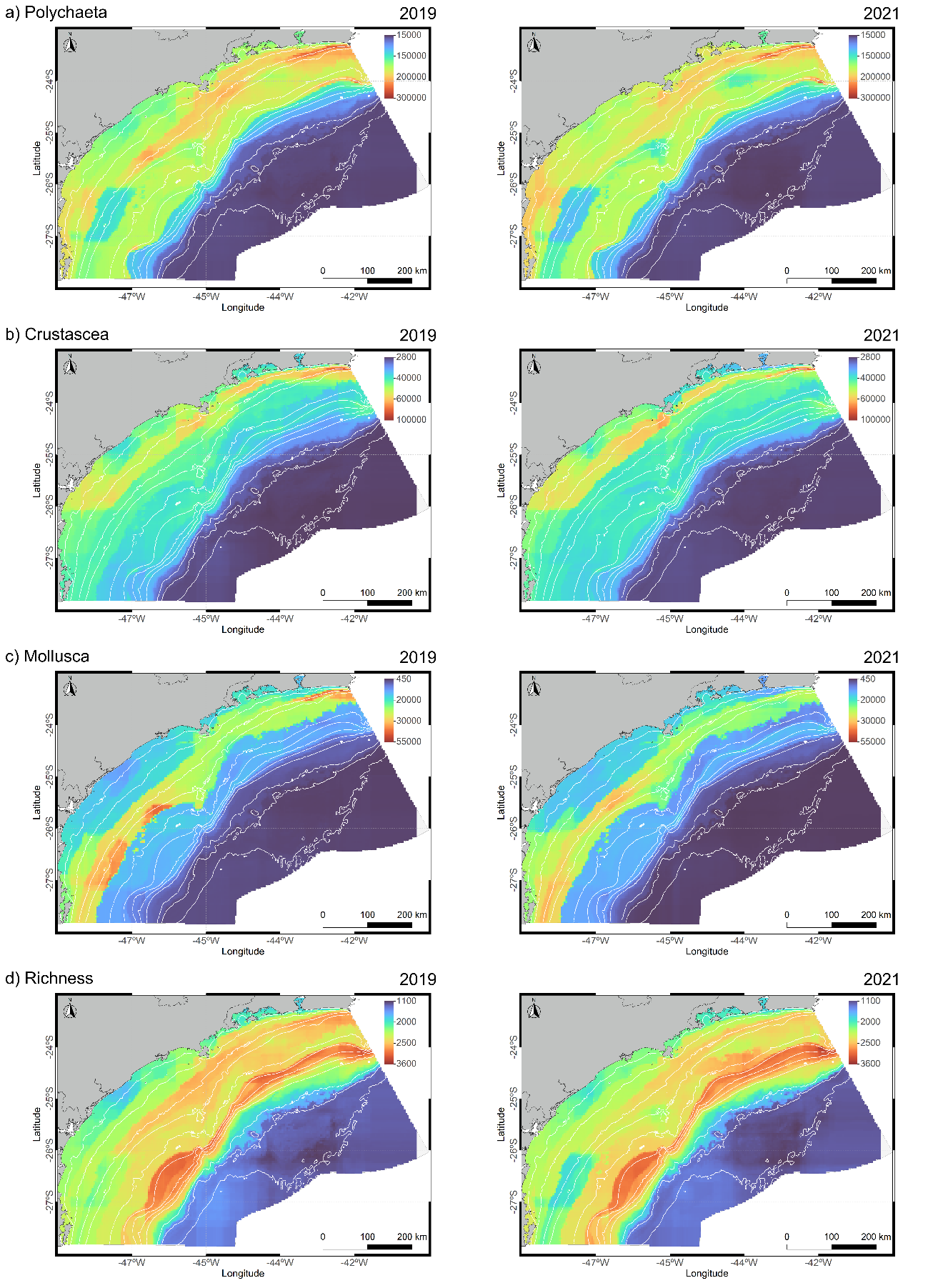


**Figure S11.** Model predictions of the Macrofauna at the Santos Basin during both sampling campaigns.

**Appendix S12**

#
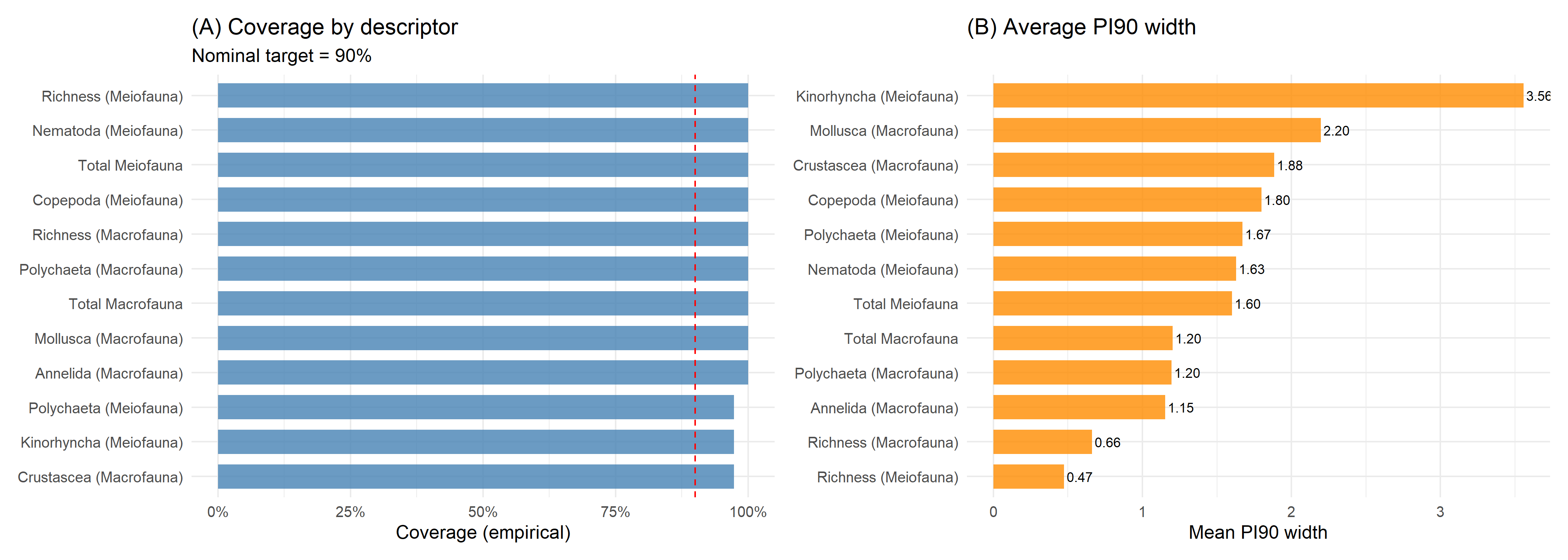


**Appendix S10**. Prediction interval coverage and average interval width across biodiversity descriptors based on the 2M models with simulated environmental predictors (2M-Sim).
(a) Empirical coverage of 90% prediction intervals (PI90) compared to the nominal target (dashed red line). (b) Mean PI90 width, showing variation among taxa.

**Appendix S13**


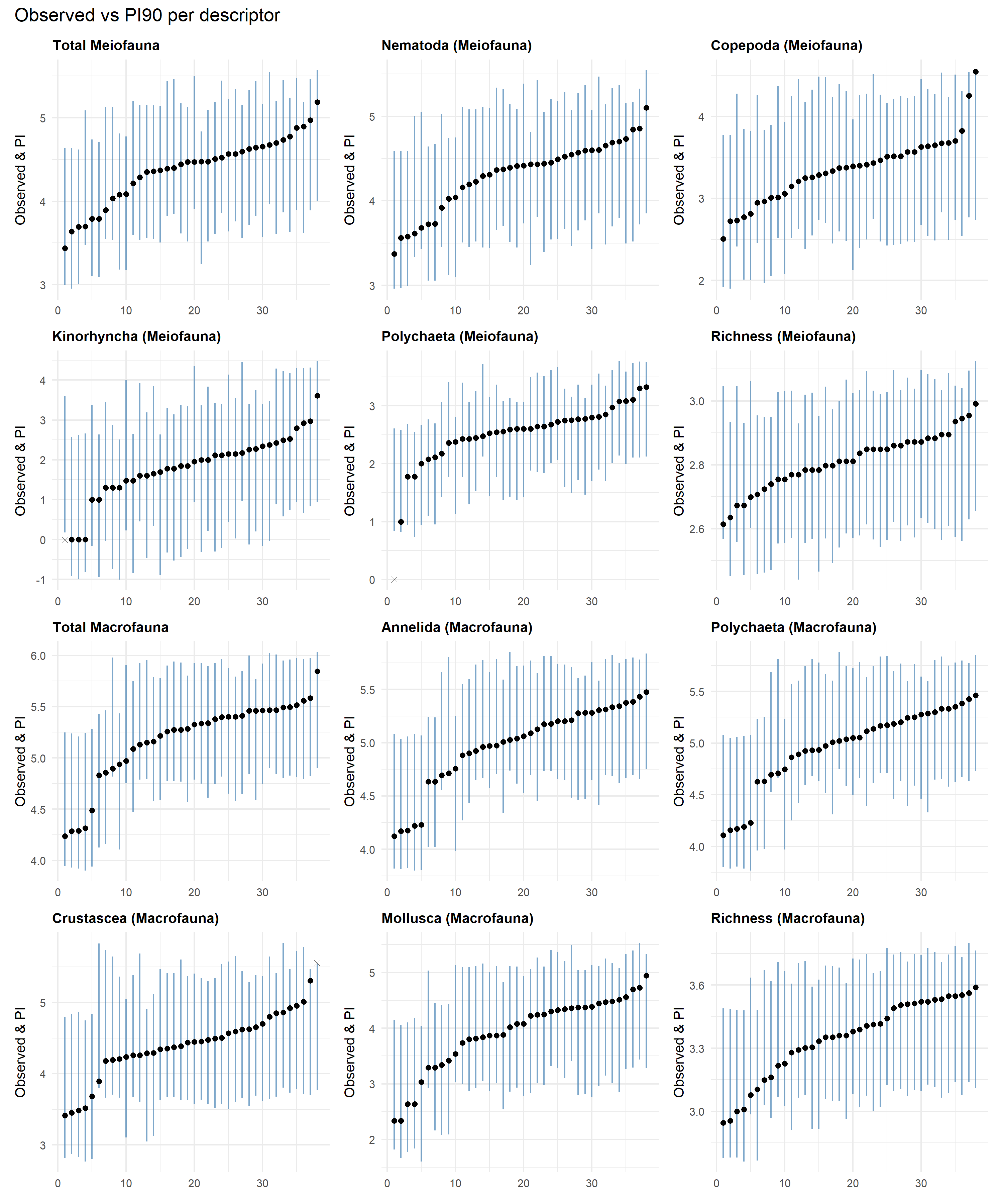


**Appendix S13.** Observed values (black dots) and 90% prediction intervals (PI90; blue bars) for each biodiversity descriptor across the validation sites (test data set). Predictions were generated using the 2M models with simulated environmental predictors (2M-Sim) combined with Monte Carlo resampling and conformal calibration.

**Appendix S14 -Instructions to Restore iMESc Savepoints**

To reproduce the analyses reported in this study, follow the steps below to restore the corresponding savepoint in iMESc:

1. If not already installed, download and install the iMESc platform following the instructions available at: https://github.com/danilocvieira/imesc
2. Download the savepoint file associated with this study from the GitHub repository (https://github.com/DaniloCVieira/imesc_savepoints/tree/1a985ef732eff5e27494ba2b64856a1c9e80870c/Fonseca_2026).
3. Open the iMESc application on your local machine.
4. Navigate to the Pre-processing tools section.
5. In the “Load a savepoint” panel:

- Click “Browse” and select the downloaded savepoint file from your computer.
-
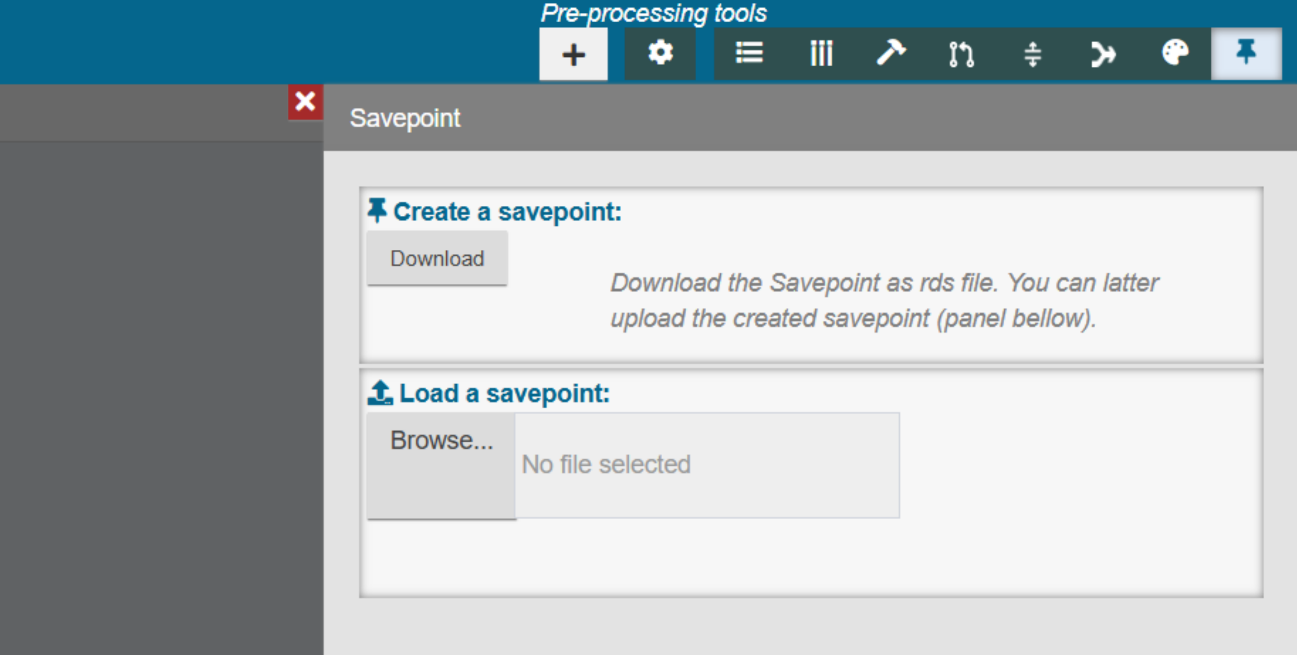
Click “Upload” (or “Load”, depending on the version) to restore the workspace.
